# Genotypic and phenotypic comparison of *Neisseria meningitidis* carriage and invasive disease isolates contemporaneously collected in the Netherlands

**DOI:** 10.1093/femsle/fnaf140

**Published:** 2025-12-16

**Authors:** Charles H Jones, Zhenghui Li, Li Hao, Arie van der Ende, Paul A Liberator, Annaliesa S Anderson, Ashlesh K Murthy

**Affiliations:** Bacterial Vaccines,Vaccines Research, Pfizer, Pearl River, NY 10965, USA; Bacterial Vaccines,Vaccines Research, Pfizer, Pearl River, NY 10965, USA; Bacterial Vaccines,Vaccines Research, Pfizer, Pearl River, NY 10965, USA; Department of Medical Microbiology, Academisch Medisch Centrum, 1100 DE Amsterdam, the Netherlands; Bacterial Vaccines,Vaccines Research, Pfizer, Pearl River, NY 10965, USA; Bacterial Vaccines,Vaccines Research, Pfizer, Pearl River, NY 10965, USA; Bacterial Vaccines,Vaccines Research, Pfizer, Pearl River, NY 10965, USA

**Keywords:** *Neisseria meningitidis*, invasive meningococcal disease, carriage, phylogenomic profile, MenB, factor H binding protein

## Abstract

*Neisseria meningitidis* (Nm), a commensal that colonizes the nasopharynx of 4.5%–24% of healthy humans, can cause invasive meningococcal disease (IMD). We hypothesized that distinct genotypic and/or phenotypic signatures might be found in carriage vs. invasive isolates. Carriage isolates were cultured from nasopharyngeal swabs (n = 267) collected from healthy students (aged 13–21 years) during the 2013 and 2014 school years in the Netherlands. Invasive isolates (n = 214) were cultured from all reported disease cases in the Netherlands from 2012 to 2014. Whole core genome sequences were determined for all isolates and comparisons of selected genotypic markers and phylogenomic associations between carriage and disease isolates were analyzed. While 30% of carriage isolates could not be assigned a genogroup, all the invasive isolates were successfully genogrouped. Genogroup B (MenB) predominated, representing 27% of carriage and 75% of IMD isolates. Sequence type (ST) complex diversity was dominated by four STs (ST-41/44, ST-213, ST-32, and ST-269) in both carriage and disease isolates. FHbp subfamily A variants were prevalent (79%) in carriage, whereas subfamily B variants were more frequent (69.6%) in disease. Carriage and IMD-causing Nm strains display similar ST and phylogenomic profiles; however, an increased FHbp subfamily B prevalence and an enhanced level of FHbp surface expression were noted in MenB disease-causing isolates.

## Teaser

*Neisseria meningitidis* isolates from two patient populations, colonized and diseased, collected during the same time period were investigated for the presence of genotypic and phenotypic markers that correlate with disease.

## Introduction

*Neisseria meningitidis* (Nm) is the causative agent of invasive meningococcal disease (IMD), in addition to frequently colonizing the upper respiratory tract in asymptomatic carriers (Gianchecchi et al. 2017). Nm presents a significant burden of disease with >400 000 cases and 32 000 deaths reported in 2019 (Shen et al. 2024), as well as associated long-term and life-altering sequelae in up to 20% of survivors (Voss et al. 2022). The incidence of disease fluctuates over time and is influenced by various factors, such as geographic location, prevailing circulating strains, and characteristics of the host population (including individuals with immune deficiencies and laboratory personnel). Additional determinants like smoking, densely populated living environments, and behaviors commonly observed in adolescents also impact disease rates (Bai et al. 2019). The incidence of IMD in North America and across Europe is 0.5/100 000 and 0.68/100 000, respectively, while in the Asia Pacific region the rates are generally between 0.02 and 0.2/100 000, with some countries, such as New Zealand, reporting significantly higher rates, 2.3/100 000 in 2019 (Aye et al. 2020). In outbreak and endemic settings, increases of 100- to 1000-fold in case rates have been reported (Bijlsma et al. 2014).

*Neisseria meningitidis* can be classified into serogroups based on the capsular structure (Purmohamad et al. 2019); of the 12 described capsule types, six serogroups (MenA, MenB, MenC, MenW, MenX, MenY) are responsible for most disease cases (Gianchecchi et al. 2017, Tzeng and Stephens 2021), with MenB strains causing the vast majority of IMD (Acevedo et al. 2019). The bacterial capsule is an important virulence factor that functions to protect the bacterium from bactericidal activity during invasion and dissemination (Unkmeir et al. 2002, Hill et al. 2010, Loh et al. 2013, Lewis and Ram 2014). Successful conjugate vaccines have been implemented for MenA (Trotter et al. 2017) or MenC (Maiden et al. 2008) individually, as well as tetravalent vaccines for MenA, MenC, MenW, and MenY, with ongoing efforts to include additional serotypes (Pizza et al. 2020). Two vaccines, Trumenba^®^ (2014) and Bexsero (2015), targeting MenB, were successfully implemented (Perez et al. 2018, Rappuoli et al. 2018) and recently followed up with approvals of Penbraya™ (2023) and Penmenvy (2024), two pentavalent vaccines covering MenA, MenB, MenC, MenW, and MenY (Petersen et al. 2023; Nolan et al. 2024).

Carriage rates of Nm in the population are estimated to be ~10% (Bijlsma et al. 2014); however, carriage rates vary with age from ~5% in infants increasing to ~24% in young adults (Christensen et al. 2010; MacLennan et al. 2021 ; Olof et al. 2022, Yue et al. 2022). Although the mechanism by which asymptomatically carried bacteria transit the mucosal epithelium leading to invasive disease is largely unknown, nasopharyngeal colonization represents the initial step in disease transmission (Gianchecchi et al. 2017). Several important large clinical studies have demonstrated the efficacy of vaccination with capsular polysaccharide-protein conjugate vaccines in reducing bacterial carriage, thus disrupting transmission (Maiden et al. 2008, Kristiansen et al. 2013). Both the MenC vaccination program in the UK and the MenA (MenAfriVac) program in Burkina Faso resulted in large impacts on carriage: a 75% reduction in MenC carriage (Maiden et al. 2008) and decrease to “undetectable” levels of MenA in parts of the African Meningitidis belt (Kristiansen et al. 2013).

Historically, Nm isolates were typed using sero-agglutination and visualization by eye (Jeppesen et al. 2015). More recently, molecular biology-based methods, RT-PCR and whole genome sequencing (WGS), have been evaluated for genogrouping Nm. WGS analysis has proven useful and robust, especially in the case of carriage isolates, for characterization and surveillance of *N. meningitidis* (Jones et al. 2016).

The present study leveraged a unique opportunity to characterize carriage and IMD isolates collected in the same geographical location across a defined time period. The contemporary two strain sets afforded the possibility of directly comparing important genetic determinants of disease in carriage and invasive disease isolates.

## Materials and methods

### Isolate collections

Two isolate collections from the same geographic region collected during the same time frame were employed to facilitate genotypic comparison of carriage strains obtained from healthy individuals vs. disease-causing isolates submitted to the Netherlands Reference Laboratory for Bacterial Meningitis (NRLBM, AMC, Amsterdam). The first study (CarMen, LI-2012, Linnaeus Institute) was designed to investigate *N. meningitidis* genogroup B carriage prevalence and acquisition rates among adolescents and young adults in the Netherlands. The study and the carriage isolates collected have been previously described (van Ravenhorst et al. 2017); briefly, students (n = 905) aged 13–21 years, covering the last 2 years of secondary school and the first 2 years of tertiary school, were enrolled into the study and provided a single nasopharyngeal (NP) swab. A subset of the students was followed longitudinally providing an additional swab at 3 and 8 months during the 2013 school year. An additional cohort of students (n = 813) was enrolled during the 2014 school year to more fully describe carriage prevalence rates (single visit) in students in the second to fifth years (aged 13–16 years) of secondary school. NP swabs were collected and processed for bacterial culture as previously described (van Ravenhorst et al. 2017). At the first visit, 270 subjects were carriage positive, yielding 267 isolates for evaluation in the study (Table 1).

**Table 1.** Subject demographics.

Age range (years)	IMD isolates, n = 214 (2012–2014)			Carriage isolates, n = 267 (2013–2014)^a^		
	MenB	Non-MenB	Total	MenB	Non-MenB	Total
0–1	47	6	53			
2–12	41	3	44			
13–21	23	7	30	72	195	267
22–50	22	9	31			
51–70	20	13	33			
71–90	7	15	22			
^a^Unknown	1	0	1			
Total	161	53	214	72	195	267

a van Ravenhorst et al. 2017.

IMD isolates were submitted to the NRLBM. The collection includes isolates obtained from ~90% of all cases during the period covering mid-2012 through the end of 2014 (Brandwagt et al. 2019) (Table 1, Supplementary Table S1). Isolates were collected predominately from blood (57%) and cerebrospinal fluid (CSF) (41%).

#### Clinical microbiology

Bacterial isolates were cultured according to standard practice as previously described (Jeppesen et al. 2015, Jones et al. 2016, van Ravenhorst et al. 2017). Briefly, following isolation of carriage isolates on modified Thayer–Martin plates (37°C, 48 hours), colonies with the appropriate morphology were subcultured on TSA II blood agar overnight at 37°C. Isolates were confirmed as *N. meningitidis* by Gram stain and oxidase testing (Oxoid, Thermo Scientific) followed by speciation through API NH tests (BioMerieux, Cambridge, MA, USA). Invasive disease isolates were cultured according to standard practice (Lam et al. 2023) and serogrouped by Ouchterlony gel diffusion (Slaterus 1961).

#### Genomic sequence analysis

Whole genome sequence analysis was conducted as previously described (Jones et al. 2016). In brief, genomic DNA was extracted from cell suspensions using magnetic bead technology (Agencourt Genfind V2; Beckman-Coulter, Indianapolis, IN, USA). DNA libraries were prepared according to the NexteraXT protocol (Illumina, San Diego, CA, USA) and genomic data obtained using the Illumina sequencing platform (MiSeq) followed by de novo assembly using the CLC Bio package (Qiagen, Germantown, MD, USA). The Bacterial Isolate Genome Sequencing Database (BIGSdb) (Jolley and Maiden 2010) was utilized to scan assembled contigs for genes of interest, determine genogroup, and make allele assignments. Raw sequence data from the Netherlands MenB isolates sequenced in this study are available at NCBI under BioProject accession PRJNA1247431.

#### Phylogenomic analysis

*Neisseria meningitidis* raw NextGen Sequencing (NGS) data were imported into CLC Genomics Workbench using the “Illumina High-Throughput Sequencing Import” tool. Failed reads were excluded during import.

Then genomes of *N. meningitidis* were assembled using the “De Novo Assembly” tool with default parameters. Alignment of the genomes was performed by Parsnp (Treangen et al. 2014) with recombination filtration enabled and with a reference genome randomly selected from the input. The final phylogenomic tree was visualized using a custom-built Java program.

The PubMLST *Neisseria* genome database was accessed on 15 July 2025. Search criteria for the isolates were invasive or disease-causing *N. meningitides* serogroup B isolates, country of origin the Netherlands, Belgium, or Germany, with collection year 2019 or later. The search resulted in 277 contemporary invasive MenB isolates from the Netherlands and Germany downloaded from the PubMLST. Netherlands MenB genomes sequenced in this study and contemporary MenB genomes downloaded from PubMLST utilizing Parsnp were used to construct a phylogenomic tree. The genome of strain MC58 served as a reference for building the tree.

### Meningococcal Antigen Surface Expression assay

Expression of FHbp was assessed using fluorescence activated cell sorting (FACS) as previously described (McNeil et al. 2009). Briefly, MenB strains were grown to mid-log, fixed with paraformaldehyde (1%), and exposed to anti-LP2086 monoclonal antibodies for 30 minutes on ice. Following two wash steps (pellet and resuspension), biotinylated goat anti-mouse IgG or IgM was added and incubated on ice for 30 minutes. After two additional wash steps (pellet and resuspension), streptavidin-PE (BD Science) was added and incubated on ice for 30 minutes. Following two final wash steps, the cell pellets were resuspended in 1% paraformaldehyde and scanned on a BD LSR II flow cytometer (20 000 events acquired). Data were analyzed using FlowJo v7 software (Treestar, Ashland, OR, USA). Isolates with a mean fluorescence intensity (MFI) >1000 were considered to be susceptible to killing in the serum bactericidal assay (McNeil et al. 2018).

## Results

### Study population

The carriage study was conducted in secondary and tertiary school students, accounting for a narrow age range of 13–21 years (van Ravenhorst et al. 2017). On the other hand, the invasive disease isolates were collected from all diagnosed patients and submitted to the national reference lab (NRLBM), representing a broad range: <1 to >90 years of age (Table 1). The ratio of females to males was biased towards females in the carriage study (62:38) (van Ravenhorst et al. 2017), with a more balanced population for the IMD subjects (51:49).

### Characterization of isolates

**Figure 1.**
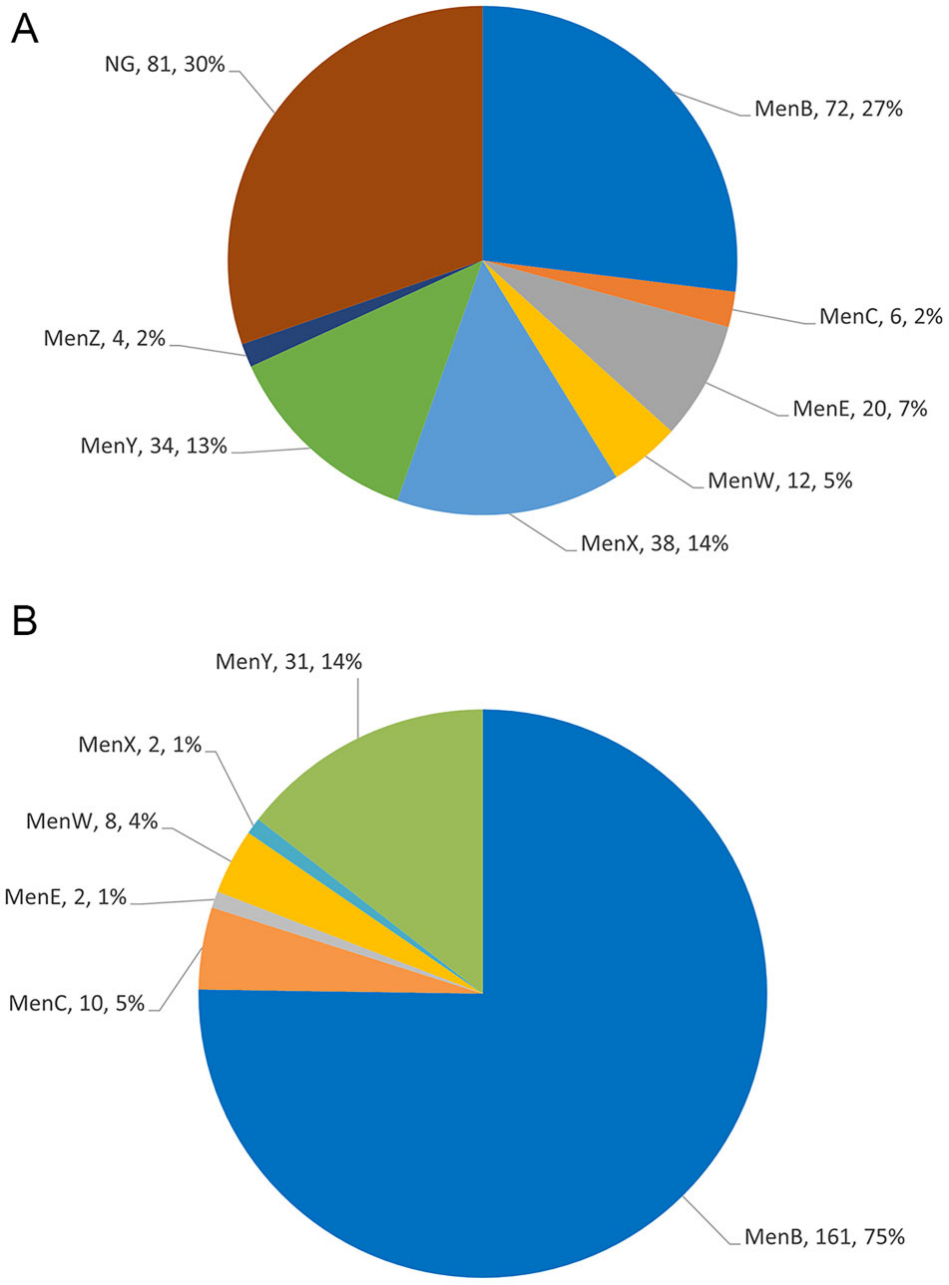
Genogroup distribution in NLS carriage (**A**, n = 267) and IMD (**B**, n = 214) isolates. Each segment of the pie chart is labeled with the genogroup, number, and percentage of isolates encoding that serogroup as determined by sequence analysis (NG: non-groupable).

Bacterial isolates from both carriage and IMD were submitted for WGS analysis to investigate genomic markers associated with pathogenesis and to characterize the collections in more detail. The genogroup distribution in each collection was determined (Fig. 1). Whereas a discrete genogroup was identified for all invasive disease isolates, 30% of carriage isolates were found to be "null" for the capsule locus (van Ravenhorst et al. 2017). This level of non-groupable isolates in carriage has been previously described (Jones et al. 2016). Genogroup B (MenB) was the predominant genogroup among groupable isolates in both isolate collections, representing 75% of IMD and 27% of carriage isolates. The MenY genogroup was well represented, 14% and 13%, respectively, in the disease and carriage strain sets. MenW strains were seen at similar levels, ~4%–5%, in both isolate collections. The MenE and MenX genogroup strains were seen at 7% and 14%, respectively, in the carriage collection; whereas these genogroups were only found at ~1% in IMD isolates, although recent outbreaks of MenX-related disease have been reported, especially in sub-Saharan Africa (Tzeng and Stephens 2021). MenC was detected at low levels, 2% and 5%, in carriage and IMD strain sets, respectively; in the Netherlands, MenC vaccination rates exceeded 90% before and during the study period (Tin Tin Htar et al. 2020).

The genogroup distribution of the disease isolates was also evaluated based on age because the IMD isolates were collected from subjects with a broad age range (<1 to >90 years) relative to the carriage collection (13–21 years). Although the numbers were small, no trends for genogroup distribution that correlated with subject age were noted (data not shown). As MenB was the predominant groupable isolate in IMD and carriage individuals, the remainder of the study focused on evaluation of MenB isolates.

**Figure 2.**
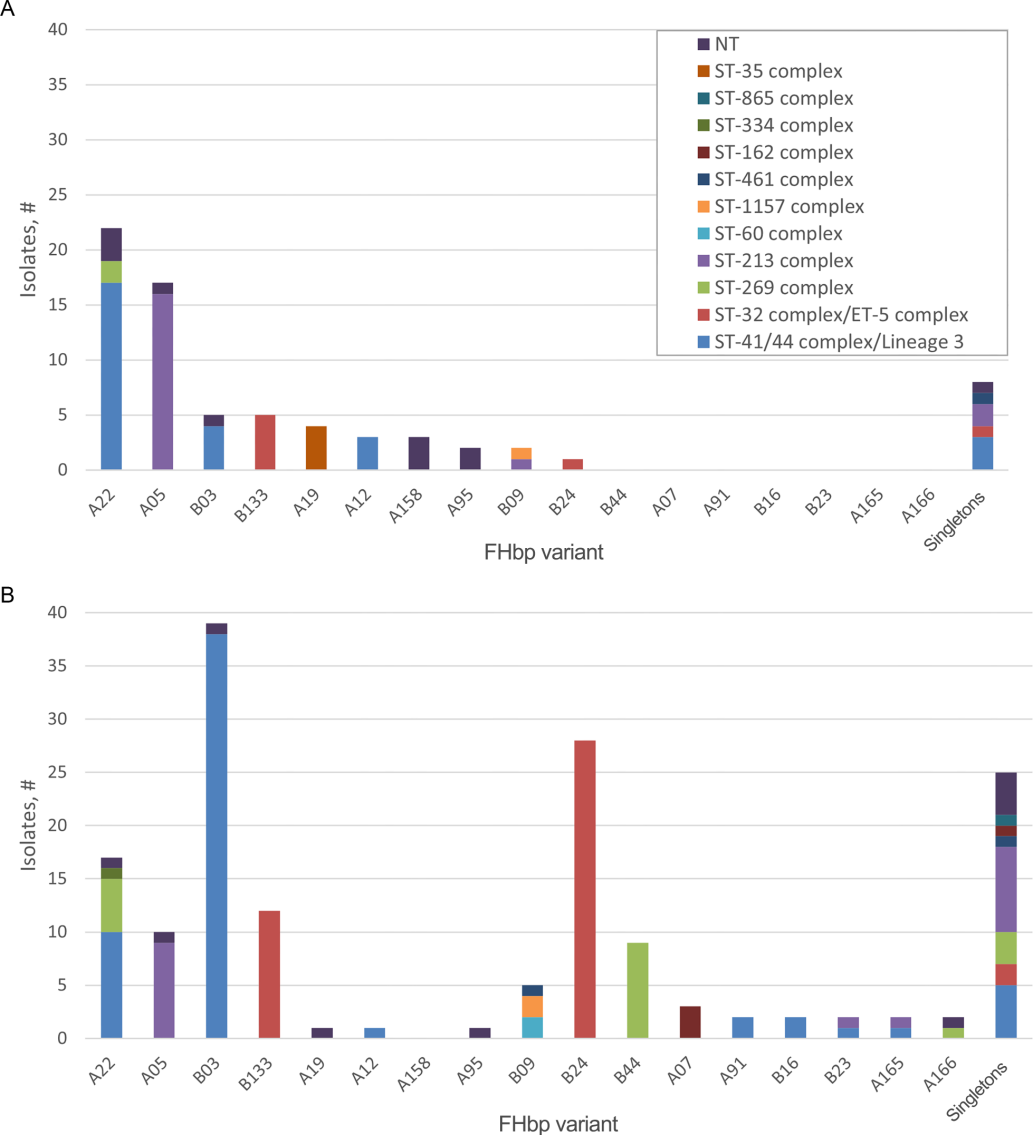
Distribution of FHbp and ST complex in carriage (**A**, n = 72) and IMD (**B**, n = 161) MenB isolates. FHbp variants common to carriage and IMD strain collections are displayed on the x-axis, with stacked bars, indicating the number of isolates in each strain set, filled according to the ST type of the isolates encoding each FHbp variant.

Four clonal complexes (CC) covered the largest part (83%) of MenB isolates. Of 233 (72 from carriage and 161 from disease) MenB isolates, 87 (37%), 38 (16%), 49 (21%), and 20 (9%) were of ST-41/44 complex/Lineage 3, ST-213 complex, ST-32 complex/ET-5 complex, and ST-269 complex, respectively (Fig. 2, Supplementary Figure S1).

The distribution of CC (Brehony et al. 2007) among disease and carriage MenB isolates was similar (Fig. 2, Supplementary Figure S1). The one exception was ST-35 complex isolates that were only seen in the carriage set. Differences in the overall percentage of isolates for the ST-213 complex, ST-32 complex/ET-5 complex, and ST-269 complex isolates were seen when comparing the two collections.

### FHbp variant distribution among MnB isolates

The distribution of FHbp variants among carriage and disease MenB isolates was dissimilar. Of the 161 MenB IMD isolates, 112 (69.6%) belonged to subfamily B (sfB), while of the 72 MnB carriage isolates, 57 (79.2%) belonged to subfamily A (sfA) (Fig. 2, Supplementary Figure S2). More specifically, FHbp A22 and A05 variants accounted for 54.2% (30.6% and 23.6%, respectively) of MenB carriage isolates, whereas the proportion of these two variants was 16% (10% and 6%, respectively) among the disease isolates. By contrast, the proportion of FHbp variants B03 (24%), B24 (17%), and B44 (6%) was higher among disease isolates than among isolates from carriers. The FHbp B133 variant is present in both the carriage and isolate collection at nearly the same level (~6%); similarly, the FHbp variant B09 appears at a low level in both the carriage and IMD strain collections; however, the MLST complexes represented differ in the two groups of strains.

Since the IMD isolates were from patients ranging in age from <1 to >90 years and the carriage collection was restricted to subjects between the ages of 13 and 21 years, the FHbp variant distribution of the disease isolates was also evaluated by age. The distribution of sfA and sfB among IMD isolates from adolescents (13–21 years old) was identical to that among IMD isolates from all patients (Table 2). Although the numbers were small, no trends for FHbp variant distribution that correlated with subject age were noted except in the case of B133 and B09, which trended higher in the 13–21 years age group (Supplementary, Figure S2).

**Table 2.** FHbp subfamily distribution in MenB carriage and IMD isolates

Group	Subfamily A (%)	Subfamily B (%)
Carriage	57 (79.2)	15 (20.8)
IMD (13–21 years)	7 (30.4)	16 (69.6)
IMD all	49 (30.4)	112 (69.6)

**Figure 3.**
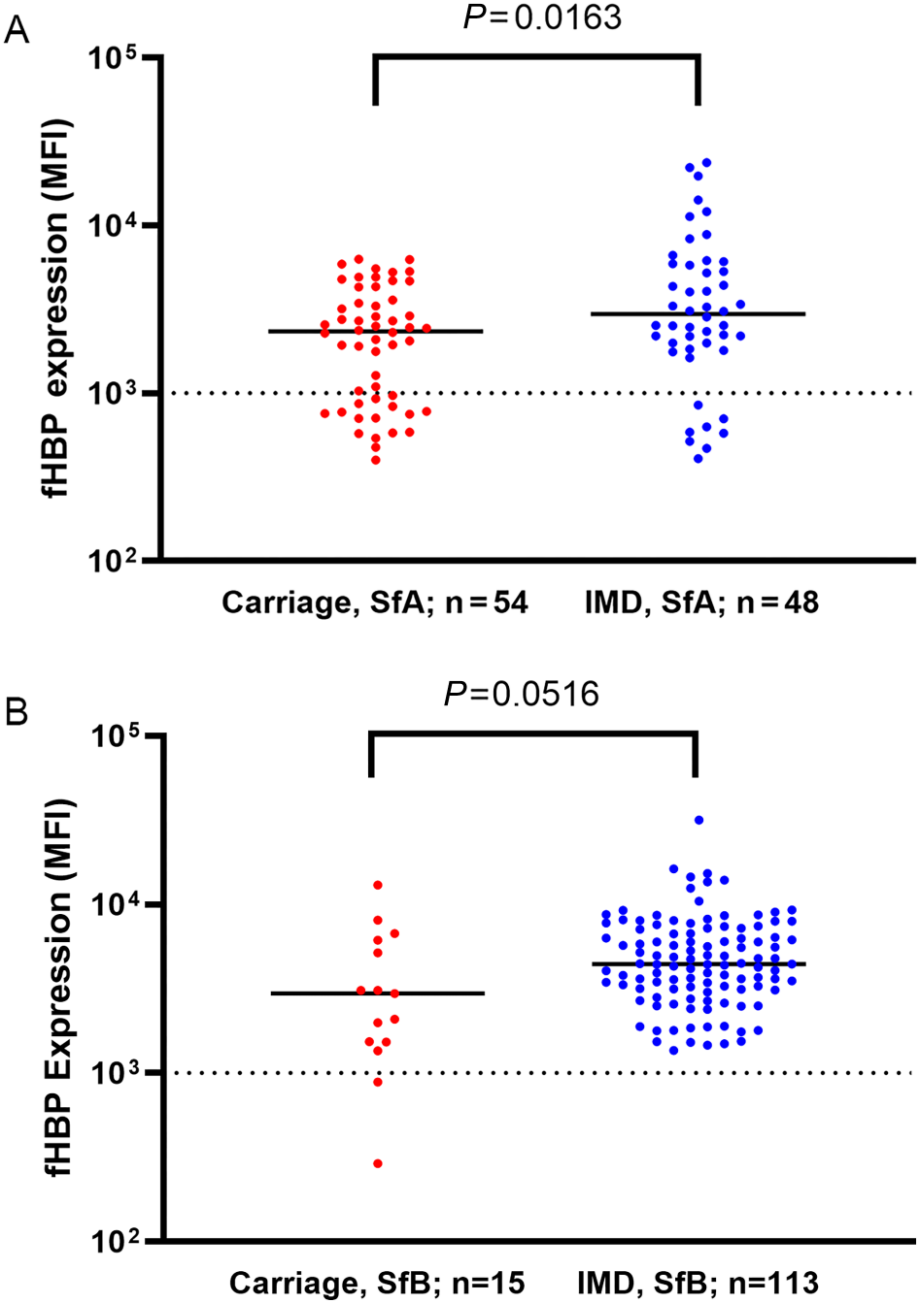
FHbp surface expression levels in subfamily A **(A)** and subfamily B **(B)** MenB isolates. Expression of FHbp in carriage (blue circles) and IMD (red circles) isolates was determined for subfamily A **(A)** and subfamily B **(B)** isolates in the MEASURE assay through recognition of FHbp by cross-reactive mAb, MN86-994-11–1 (McNeil et al. 2018). Mean fluorescence intensity (MFI) is presented on the y-axis. Significance is presented as *P* values from Welch’s unpaired t-test.

Surface expression of FHbp on carriage and IMD isolates was assessed by FACS (Fig. 3). The Meningococcal Antigen Surface Expression (MEASURE) assay was developed to quantify surface expression levels of FHbp on MnB isolates and to correlate those with susceptibility to bactericidal killing in serum (McNeil et al. 2018). All 113 IMD and 13 (87%) carriage isolates with sfB FHbp and 40 (83%) IMD isolates with sfA FHbp had an MFI >1000, a threshold of surface FHbp expression that correlates with a high probability of bactericidal activity in the serum bactericidal assay (Fig. 3b). Conversely, 16 (30%) carriage isolates with sfA FHbp had an MFI <1000, which correlates with a lower probability of serum bactericidal activity. Interestingly, 4/5 of the IMD isolates with sfB FHbp with the highest MFI belonged to the ST-269 complex, while 5/5 of the carriage isolates with sfB FHbp with the highest MFI were from the ST-32 complex/ET-5 complex (data not shown). In the case of the subfamily A isolates, there was not a clear correlation with MLST complex and level of FHbp expression.

**Figure 4.**
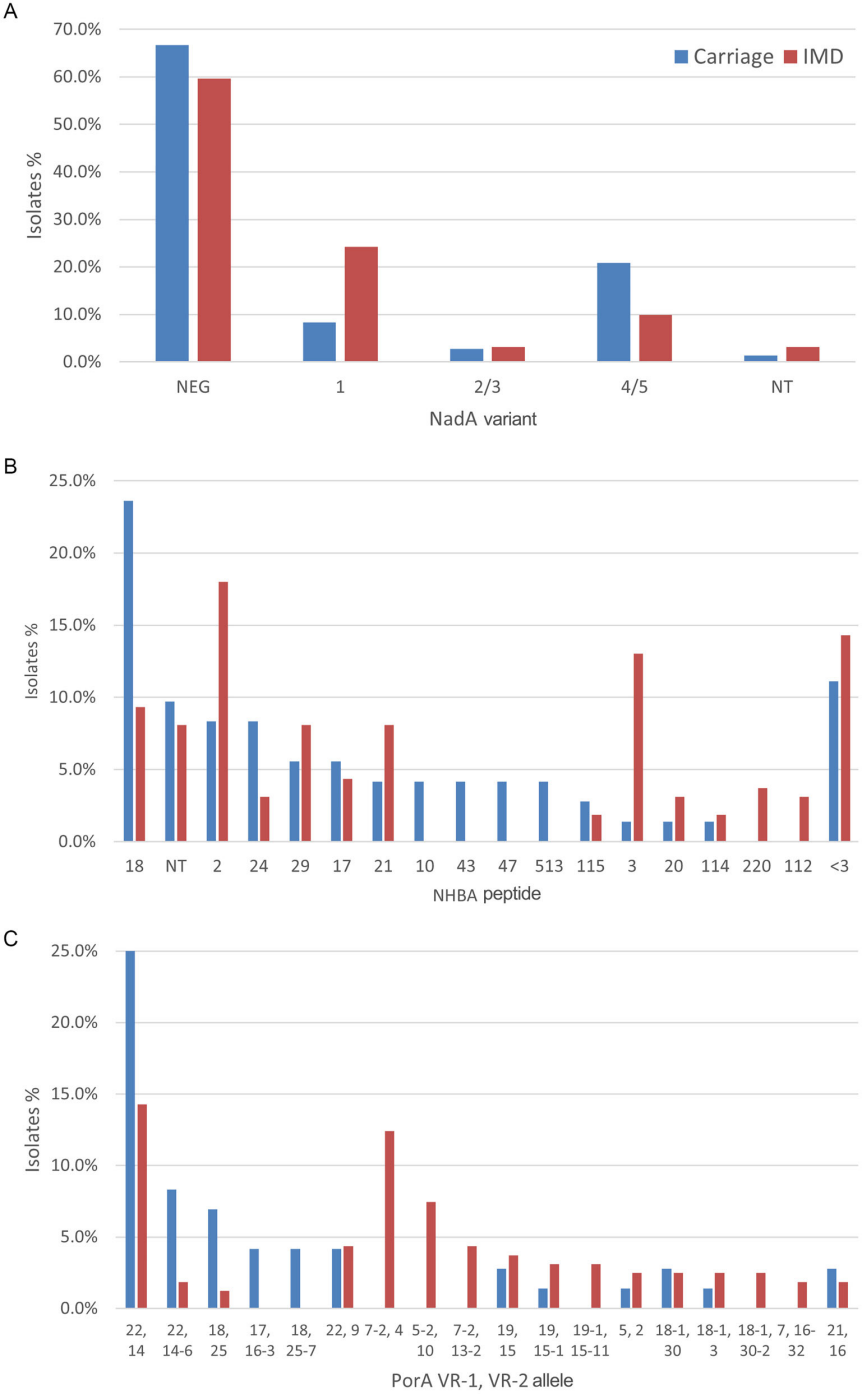
Presence of additional antigens of interest in carriage (n = 72) and IMD (n = 161) MenB strains. NadA variant **(A)**, NHBA peptide **(B)**, and PorA VR-1, VR-2 alleles **(C)** were determined by sequence analysis for MenB carriage (blue bars) and IMD (red bars) isolates.

In addition to FHbp, NadA and NHBA are included in 4CMenB (Bexsero™), which also includes the PorA variant 7–2, 4 contained in the outer membrane vesicle (OMV) component of the vaccine (Lucidarme et al. 2009, Granoff 2010, Gorringe and Pajón 2012, Carter 2013). Of the MenB isolates from patients and carriers, 60% and 68%, respectively, did not encode a NadA variant and <5% encoded the 2/3 variant present in 4CMenB (Fig. 4a). NHBA peptide variant 18 was dominant among MenB isolates from patients, while variants 2 and 3 were more present among carriage isolates (Fig. 4b). The variation of PorA VR-1, VR-2 alleles was large. However, only 12% of the MenB IMD isolates encoded for the PorA variant 7–2, 4, while it was absent among carriage isolates; this VR-1, VR-2 allele combination is one of many found in the two strain collections (Fig. 4c).

### Phylogenomic characterization of isolates

**Figure 5.**
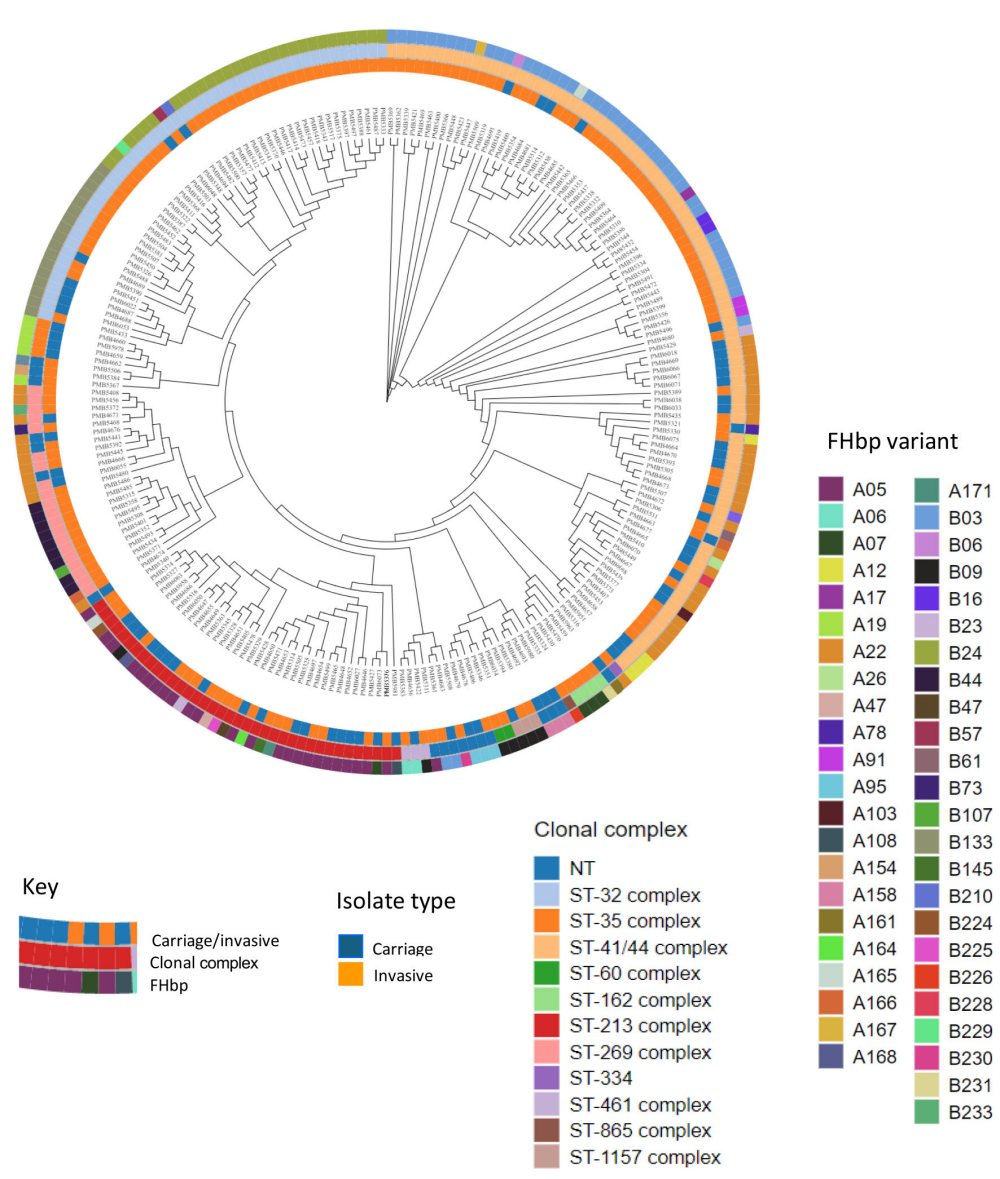
Phylogenomic analysis of MenB carriage (n = 72) and IMD (n = 161) isolates. Phylogenomic tree of the MenB isolates characterized in this study. From the inner ring to the outer ring, the phylogenomic tree is color coded by isolate type (carriage vs. invasive), clonal complex (ST), and FHbp variant. Each branch represents the genome of a MenB isolate.

Core genome phylogeny was assessed to detect any groupings among carriage MenB isolates and MenB isolates from patients (Fig. 5). The clustering of the genomes is driven by CC. Overall, within CC, carrier and disease isolates intermingled with the exception of ST-35 complex, which exclusively consisted of carriage isolates. Within each cluster, both subfamily A and subfamily B FHbp variants are observed. From the 12 o’clock position going clockwise, a large number of isolates that are ST-41/44 complex/Lineage 3 (N = 87) formed a cluster with a few non-typeable exceptions. In this cluster, the most frequently observed FHbp variants are B03 (N = 42) and A22 (N = 27). From 6 o’clock to 7 o’clock, isolates that are ST-213 complex (N = 38) formed a cluster with the majority of the isolates FHbp A05 (N = 25). From 10 o’clock to 12 o’clock, isolates that are ST-32 complex/ET-5 complex (N = 49) formed a cluster. In this cluster, the most frequent FHbp variants are B24 (N = 29) and B133 (N = 17) with the remaining FHbp variants as singletons.

Investigating the relationship between clonal complex and FHbp variant (Supplementary Figure S3) demonstrated trends with differing proportions of FHbp variants associated with specific CC in carriage and disease; for example, 63.3% of ST41/44 complex/Lineage 3 isolates in disease are B03, with just under 17% A22. Conversely, in carriage, 63% of ST41/44 complex/Lineage 3 isolates are A22 with just under 15% B03. Similarly, a higher percentage of ST32 complex/ET-5 disease isolates are B24 (87.5%) compared with ~14% of carriage isolates, and the opposite is seen for B133, with 28.6% of disease isolates and 71.4% of carriage isolates having the B133 variant.

### Contemporary MenB isolates

Utilizing genomes accessed from PubMLST covering contemporary strains, collected 2019–2022 from the Netherlands (n = 94) and Germany (n = 183), a combined phylogenomic tree was constructed (Supplementary Figures S4, S5). The PubMLST genomes are interspersed throughout the phylogenomic tree and there is no delineation between the Netherlands MenB genomes sequenced from the present study and the more contemporary genomes from PubMLST. Clustering of the strains is driven by clonal complex with the predominate CC for the older strain set and the contemporary strains: ST-41/44 complex Lineage 3 (37% vs. 32%), ST-32 complex/ET-5 complex (26% vs. 22%), ST-213 complex (12% vs. 19%), and ST-269 complex (11% vs. 12%). Within the predominate CC, the FHbp variant distributions of the PubMLST genomes were aligned, with the most prevalent FHbp variants seen in the study isolates (Supplementary Figure S5). For example, ST-41/44 complex/Lineage 3 was associated predominately with FHbp variants A22, B03, and B16 for both the study isolates as well as the PubMLST genomes. Similarly, ST-32 complex/ET-5 complex was associated with B133 and B24 for both collections.

## Discussion

Nasopharyngeal colonization represents the initial step in disease transmission for the dedicated human pathogen, Nm. Discrete meningococcal factors that predispose Nm to asymptomatic nasopharyngeal colonization vs. causing invasive disease have yet to be identified (Gianchecchi et al. 2017, Eriksson et al. 2023). Utilizing *N. meningitidis* carriage and invasive disease strain collections obtained from the same location during the 2012–2014 time frame, genomic factors that could potentially differentiate carriage and disease isolates were investigated in this study. The identification of non-groupable strains and ST-35 complex exclusively in carriage isolates was notable. However, capsule null strains have been implicated in disease for both immunocompetent and immune-compromised individuals (Hoang et al. 2005, Findlow et al. 2007, Tzeng and Stephens 2021). In addition, an increased FHbp subfamily B prevalence and an enhanced level of FHbp surface expression were seen in MenB disease-causing isolates; however, no unique signatures of IMD isolates were identified.

Prior efforts to investigate endemic and epidemic Nm clones, such as W cc11 (Hajj clone), have been described (Mustapha et al. 2016); however, sequencing of W cc11 clones revealed that sporadic case clusters of W cc11 disease arose in the meningitis belt, Brazil, the UK, and Taiwan prior to the 2000 Hajj outbreak (Mustapha et al. 2016). These findings suggest that W cc11 consists of several genetically and geographically diverse sub-lineages (Mustapha et al. 2016). Sequence analysis further indicated that W cc11 arose following capsular switching and clonal expansion along with additional recombination events that resulted in acquisition of novel virulence determinants (Buckee et al. 2008, Kong et al. 2013). The precise genetic and environmental factors that resulted in the development of the Hajj clone have yet to be fully described.

In the present study, although MenB represented 75% of all the invasive disease isolates and 27% of the carriage isolates in the study, the other major endemic serotypes (MenC, MenW, MenX, MenY) associated with invasive disease were also present in carriage isolates. Whereas all of the IMD isolates had a discrete capsule type assigned, 30% of the carriage isolates were found to be capsule null (*cnl*), as previously described (van Ravenhorst et al. 2017). A report by Neri et al. (2019) described Nm carriage in Italian high school students and reported that 47% of isolates were *cnl* and most often associated with ST-198 complex and ST-1136 complex and, to a lesser degree, with ST-53 complex, ST-213 complex, ST-32 complex/ET-5 complex, ST-41/44 complex/Lineage 3, and ST-1117 complex. Non-groupable and *cnl* Nm have been reported to cause invasive disease in immune-compromised as well as immunocompetent individuals and more recently have been associated with sexually transmitted urethritis cases (Hoang et al. 2005, Findlow et al. 2007, Tzeng and Stephens 2021, Rodriguez et al. 2023).

WGS data analysis revealed that MLST-type distribution was similar between carriage and invasive disease isolates. Looking specifically at MenB isolates from both collections, 77% and 86% of carriage and IMD isolates, respectively, were represented by four ST complexes: ST-41/44 complex/Lineage 3, ST-213 complex, ST-32 complex/ET-5 complex, and ST-269 complex (Fig. 2, Supplementary Figure S2). A small number of ST-35 complex isolates were unique to the carriage set, and ST-60 complex, ST-865 complex, and ST-334 complex isolates were found only in the IMD set; these outliers may simply be a result of a small representation of the specific STs. In agreement with the Neri et al. (2019) study, 78% of the *cnl* carriage isolates belonged to one of the following CC: ST-53 complex, ST-41/44 complex/Lineage 3, ST-198 complex, ST-1136 complex, or ST-1117 complex. These findings underlie that most common strain lineages of Nm are found both circulating in the community as well as in invasive disease.

WGS data analysis further revealed that the distribution of FHbp variant types was similar between carriage and invasive disease isolates. The current finding supports previously published data (Murphy et al. 2009, Anderson et al. 2013, Li et al. 2024) demonstrating that in disease collections isolates expressing subfamily B FHbp are more prevalent (69%), whereas in carriage collections the converse is true, with isolates expressing subfamily A FHbp showing a higher prevalence (79%). In line with this finding, A22 and A05 variants were found in significantly higher numbers among the carriage isolates and B03, B24, and B44 variants more prevalent in the disease isolates. Although fewer in number, A22 and A05 variants were found in the disease collection and B03 and B24 variants were identified in the carriage strain set. FHbp B44 variant was found only in disease isolates; however, the total number of B44 isolates obtained was small.

A phylogenomic tree was constructed to better demonstrate the relationship between carriage and invasive disease isolates based on the core genomes. Carriage and IMD isolates were intermingled on the phylogenomic tree and occupied nearly every portion of the tree. The clustering of the core sequences on the tree is driven by clonal complex (ST). FHbp variants, sourced from both carriage and invasive disease, were found amongst each cluster. The segment around 11 to 2 o’clock is the only region that is populated predominantly with invasive disease isolates, which reflects the importance of the MenB ST-41/44 complex/Lineage 3 and ST-32 complex/ET-5 complex isolates in disease (Brehony et al. 2007), although in both cases carriage isolates are co-located in the cluster. Furthermore, a trend was noted for FHbp variants to associate with CC (Supplementary Figure S3); specifically, B03 and B24 in ST41/44 complex/Lineage 3 and ST32 complex/ET5 disease isolates, respectively, and A22 and B133 in ST41/44 complex/Lineage 3 and ST32 complex/ET5 carriage isolates, respectively. Larger datasets will need to be investigated to confirm this trend.

A perceived limitation of our study could be the age of the isolates (collected 2012–2014), such that the findings may not reflect current epidemiology in the Netherlands. Utilizing genomes accessed from PubMLST covering contemporary strains (post-2019 collection) from the Netherlands and Germany, a combined phylogenomic tree was constructed (Supplementary Figure S4). Based on the phylogenomic tree, there is no clear separation between the Netherlands MenB genomes sequenced from the present study and the more contemporary genomes from PubMLST, suggesting that the age of the isolates in this study does not adversely impact our interpretations. Clustering of the strains is driven by clonal complex primarily; for example, all ST-32 complex isolates clustered together irrespective of time of collection or country of origin. In addition, for the contemporary genomes, the predominate CCs (ST-41/44 complex/Lineage 3, ST-32 complex/ET-5 complex, ST-213 complex, ST-269 complex) mirrored those seen with the study isolates and further, the composition of FHbp variants associated with each clonal complex was aligned between the two data sources. These findings suggest that the carriage and IMD isolates collected in the original study (2012–2014) are an accurate reflection of the current epidemiology of Nm in the Netherlands and, further, that in the Netherlands the predominate Nm strains have been maintained to a large degree over the last two decades.

A curious finding concerns the expression levels of FHbp, which appear to be elevated in isolates expressing subfamily B (sfB) variants, especially in invasive disease isolates, which was reported previously (Lemée et al. 2014). Early work to develop a quantitative assay to evaluate FHbp expression *in vitro* identified an expression level (1000 MFI) that was predictive of probability of bacterial killing in the human serum bactericidal assay (hSBA) (McNeil et al. 2018); however, the hSBA revealed a wide range of expression levels for Nm isolates *in vitro*. Work in the Tang lab demonstrated thermoregulation of FHbp expression due to an upstream riboswitch (Loh et al. 2016) responsive to the temperature gradient in host tissues: low temperature, low level FBbp expression in the pharyngeal region, and high temperature, elevated expression of FHbp in the blood. Additional environmental factors have been shown to influence FHbp expression, including both oxygen and iron levels (Oriente et al. 2010, Yee et al. 2023).

Earle et al. (2021) conducted a bacterial genome wide association study of 1556 Nm and identified single-nucleotide polymorphisms associated with invasive disease vs. carriage in several loci across the genome underlying the polygenic nature of IMD. One significant peak of association was located in the intergenic region between the *fba* (*cbbA*) and *FHbp* open reading frames. A polymorphism at FHbp S-7 T/C was predicted to impact the local RNA structure around the ribosome binding site such that it was more open and accessible. This variant conferred higher FHbp expression and increased serum survival, both of which are proposed to translate *in vivo* to enhanced resistance to complement mediated killing. This association was first noted in ST-11 complex strains and replicated in ST-41/44 complex/Lineage 3 strains, suggesting a generalized mechanism for controlling FHbp expression. Taking a different approach, Spinsanti et al. (2021) inspected the FHbp intergenic region (fIR) from >5800 strains and identified nine dominant fIR alleles that produced distinct levels of FHbp, thus demonstrating that the fIR region determines the level of FHbp expression. Furthermore, this group then looked at a panel of UK strains and observed that strains with a higher expression level of FHbp were “more likely” to be associated with invasive disease. Lastly, the fIR alleles were found to associate with specific CC, with fIR alleles dictating higher expression levels in CC correlated with IMD (ST-41/44 complex/Lineage 3, ST-32 complex/ET-5 complex) (Spinsanti et al. 2021).

In summary, this study presented a unique opportunity to inspect carriage and disease-causing *N. meningitidis* isolates, acquired during a discrete time period, at the genomic level. The large percentage of non-groupable isolates in the carriage set and an increased subfamily B prevalence in the disease set were notable differences; however, the ST and phylogenomic profiles were similar and a unique signature for disease isolates was not identified other than the capsular polysaccharide geno- and pheno-types. As the present study demonstrates, the expression level of FHbp, which trends higher in isolates associated with disease, may be an important factor in the transition from asymptomatic carriage to invasive disease. The maintenance of FHbp during carriage may underlie the transition to an IMD-causing isolate through modulation of expression levels. Whereas unique genotypic signatures of virulence were not identified, the data suggest a possible contribution of multiple bacterial virulence determinants. Furthermore, a possible role for host susceptibility factors in the transition to disease is suggested and supports the importance of vaccines that induce an antibody response against the vast array of FHbp variants produced by Nm. Importantly, the lack of clearly identifiable signatures strengthens the case for preventative vaccination, as opposed to targeted interventions, as the mainstay to protect against morbidity and mortality caused by IMD.

## Supplementary Material

fnaf140_Supplemental_Files
