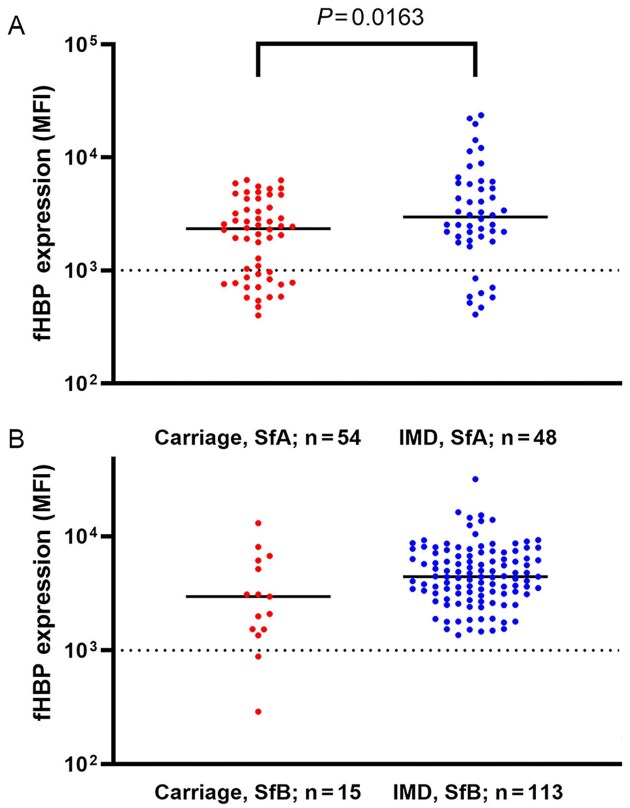# Correction to: Genotypic and phenotypic comparison of *Neisseria meningitidis* carriage and invasive disease isolates contemporaneously collected in the Netherlands

**DOI:** 10.1093/femsle/fnag081

**Published:** 2026-07-14

## Abstract

We found evidence that nitrofurantoin had *in vivo* and *in vitro* activity against *Neisseria gonorrhoeae*.

This is a correction to: Charles H Jones, Zhenghui Li, Li Hao, Arie van der Ende, Paul A Liberator, Annaliesa S Anderson, Ashlesh K Murthy, Genotypic and phenotypic comparison of *Neisseria meningitidis* carriage and invasive disease isolates contemporaneously collected in the Netherlands, *FEMS Microbiology Letters*, Volume 373, 2026, fnaf140, https://doi.org/10.1093/femsle/fnaf140

In the originally published version of this manuscript, the p-value for Figure 3B and the top most marker on the y-axis were inadvertently cut off.

Figure 3 has been corrected to read:

**Figure:**
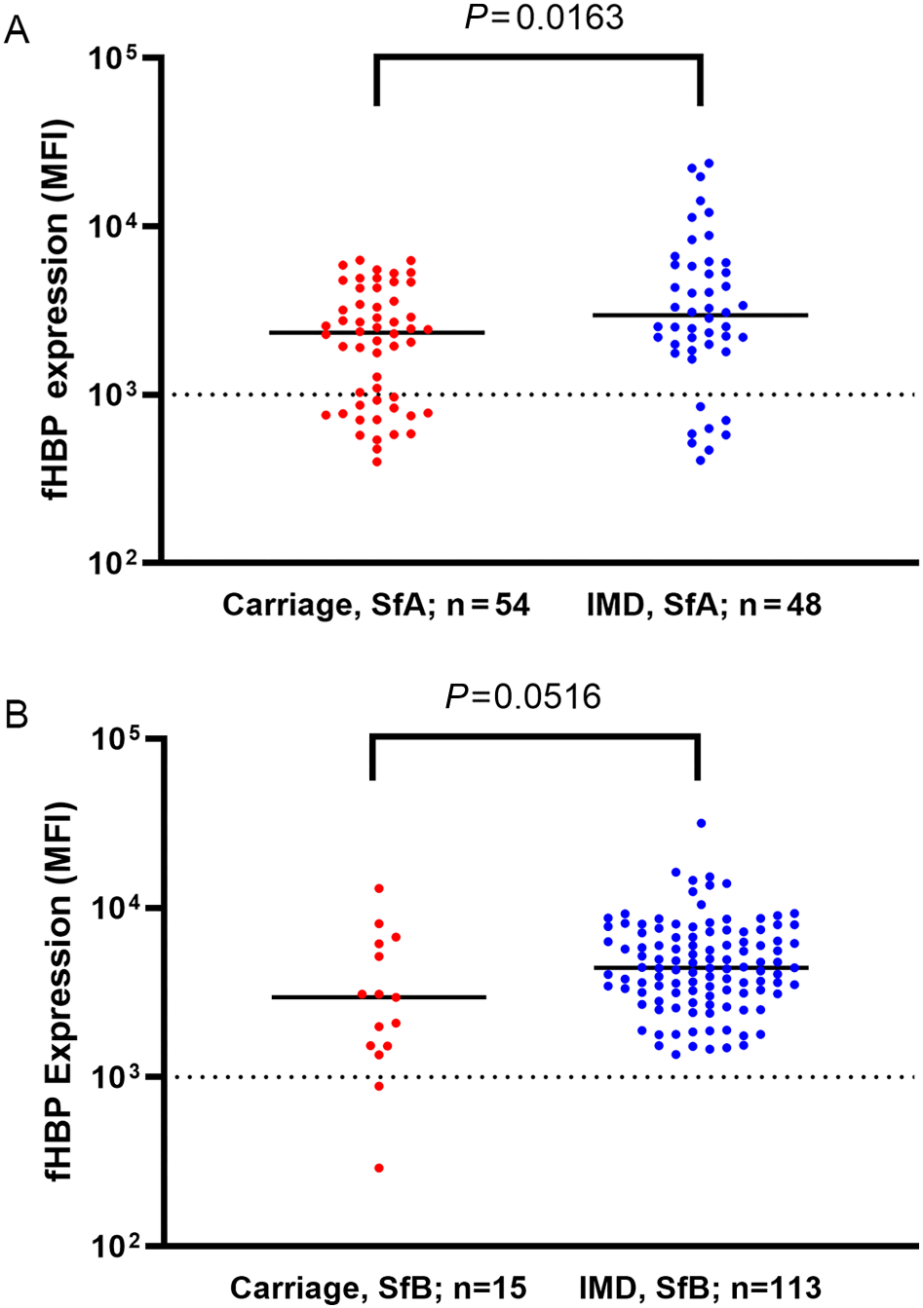


Instead of:

**Figure:**