# Supplementary material for: Genotypic and phenotypic comparison of Neisseria meningitidis carriage and invasive disease isolates contemporaneously collected in the Netherlands
Source: FEMS Microbiol Lett. 2025 Dec 16;373:fnaf140. doi: 10.1093/femsle/fnaf140 (PMC12794619; doi:10.1093/femsle/fnaf140)
Supplement: fnaf140_Supplemental_Files [file fnaf140_supplemental_files.zip › NLS CARvsIMD_Supplementary Table.docx]

**Supplementary Table S1: Netherlands Invasive Disease Isolates**

| **ID** | **Age (years)** | **Gender** | **Source** | **FHbp** | **ST/Clonal complex** | **WGS GG** |
| --- | --- | --- | --- | --- | --- | --- |
| PMB5304 | 8 | M | CSF | B03 | ST-41/44 complex/Lineage 3 | MenB |
| PMB5305 | 0 | F | CSF | A22 | ST-41/44 complex/Lineage 3 | MenB |
| PMB5306 | 2 | F | CSF | B61 | ST-41/44 complex/Lineage 3 | MenB |
| PMB5307 | 80 | M | Blood | A180 | ST-41/44 complex/Lineage 3 | MenB |
| PMB5308 | 14 | M | Blood | B44 | ST-269 complex | MenB |
| PMB5309 | 9 | F | Blood | A15 | ST-23 complex/Cluster A3 | MenY |
| PMB5310 | 10 | M | CSF | B03 | ST-41/44 complex/Lineage 3 | MenB |
| PMB5311 | 3 | F | Blood | A05 | ST-939 | MenB |
| PMB5312 | 1 | M | CSF | A165 | ST-41/44 complex/Lineage 3 | MenB |
| PMB5313 | 43 | M | Blood | A10 | ST-11 complex/ET-37 complex | MenW |
| PMB5314 | 0 | F | Blood | B145 | ST-213 complex | MenB |
| PMB5315 | 23 | M | Blood | B44 | ST-269 complex | MenB |
| PMB5316 | 0 | F | CSF | A22 | ST-334 | MenB |
| PMB5317 | 59 | M | Blood | A19 | ST-5436 | MenY |
| PMB5318 | 84 | F | Blood | A26 | ST-167 complex | MenY |
| PMB5319 | 15 | M | CSF | B03 | ST-41/44 complex/Lineage 3 | MenB |
| PMB5320 | 40 | M | CSF | A22 | ST-41/44 complex/Lineage 3 | MenC |
| PMB5321 | 25 | F | Blood | A78 | ST-9552 | MenB |
| PMB5322 | 26 | M | CSF | B24 | ST-32 complex/ET-5 complex | MenB |
| PMB5323 | 0 | M | CSF | A10 | ST-11 complex/ET-37 complex | MenC |
| PMB5324 | 0 | M | CSF | A07 | ST-162 complex | MenB |
| PMB5325 | 18 | F | CSF | A05 | ST-213 complex | MenB |
| PMB5326 | 44 | M | CSF | B133 | ST-32 complex/ET-5 complex | MenB |
| PMB5327 | 17 | M | CSF | B224 | ST-213 complex | MenB |
| PMB5328 | 0 | F | CSF | B23 | ST-213 complex | MenB |
| PMB5329 | 0 | F | Blood | B225 | ST-213 complex | MenB |
| PMB5330 | 89 | M | Blood | A12 | ST-41/44 complex/Lineage 3 | MenB |
| PMB5331 | 5 | M | Blood | B102 | ST-167 complex | MenY |
| PMB5332 | 1 | F | Blood | B03 | ST-41/44 complex/Lineage 3 | MenB |
| PMB5333 | 3 | F | CSF | B24 | ST-32 complex/ET-5 complex | MenB |
| PMB5334 | 1 | M | CSF | B16 | ST-41/44 complex/Lineage 3 | MenB |
| PMB5335 | 1 | F | CSF | B226 | ST-865 complex | MenB |
| PMB5336 | 18 | M | Blood | A15 | ST-23 complex/Cluster A3 | MenY |
| PMB5337 | 1 | M | Blood | B111 | ST-23 complex/Cluster A3 | MenY |
| PMB5338 | 4 | M | CSF | B03 | ST-41/44 complex/Lineage 3 | MenB |
| PMB5339 | 25 | M | Blood | B03 | ST-41/44 complex/Lineage 3 | MenB |
| PMB5340 | 0 | M | Blood | A05 | ST-213 complex | MenB |
| PMB5341 | 53 | F | Blood | B24 | ST-32 complex/ET-5 complex | MenB |
| PMB5342 | 66 | F | CSF | A15 | ST-23 complex/Cluster A3 | MenY |
| PMB5343 | 53 | F | CSF | B24 | ST-32 complex/ET-5 complex | MenB |
| PMB5344 | 11 | F | Blood | A17 | ST-41/44 complex/Lineage 3 | MenB |
| PMB5345 | 0 | F | CSF | A05 | ST-213 complex | MenB |
| PMB5346 | 0 | M | Blood | B09 | ST-60 complex | MenB |
| PMB5347 | 80 | F | Blood | A10 | ST-11 complex/ET-37 complex | MenW |
| PMB5348 | 25 | F | Blood | B57 | ST-32 complex/ET-5 complex | MenB |
| PMB5349 | 1 | F | Blood | B227 | ST-11 complex/ET-37 complex | MenC |
| PMB5350 | 49 | F | Blood | A12 | ST-167 complex | MenY |
| PMB5351 | 4 | F | Blood | B09 | ST-60 complex | MenB |
| PMB5352 | 68 | F | CSF | B107 | ST-269 complex | MenB |
| PMB5353 | 0 | M | CSF | B03 | ST-41/44 complex/Lineage 3 | MenB |
| PMB5354 | 3 | F | Blood | B03 | ST-41/44 complex/Lineage 3 | MenB |
| PMB5355 | 77 | F | Blood | B139 | ST-11 complex/ET-37 complex | MenC |
| PMB5356 | 1 | F | CSF | B03 | ST-41/44 complex/Lineage 3 | MenB |
| PMB5357 | 4 | F | CSF | B24 | ST-32 complex/ET-5 complex | MenB |
| PMB5358 | 46 | M | CSF | B44 | ST-269 complex | MenB |
| PMB5359 | 79 | M | Blood | A15 | ST-23 complex/Cluster A3 | MenY |
| PMB5360 | 0 | M | Blood | A99 | ST-22 complex | MenW |
| PMB5361 | 2 | M | CSF | B03 | ST-13619 | MenB |
| PMB5362 | 20 | M | CSF | B03 | ST-41/44 complex/Lineage 3 | MenB |
| PMB5363 | 0 | M | CSF | A05 | ST-213 complex | MenB |
| PMB5364 | 53 | F | CSF | B03 | ST-41/44 complex/Lineage 3 | MenB |
| PMB5365 | 5 | F | CSF | B03 | ST-41/44 complex/Lineage 3 | MenB |
| PMB5366 | 1 | M | Blood | B03 | ST-41/44 complex/Lineage 3 | MenB |
| PMB5367 | 2 | F | Blood | A19 | ST-13620 | MenB |
| PMB5368 | 0 | F | CSF | B24 | ST-32 complex/ET-5 complex | MenB |
| PMB5369 | 1 | M | CSF | B03 | ST-41/44 complex/Lineage 3 | MenB |
| PMB5370 | 16 | F | Blood | B24 | ST-32 complex/ET-5 complex | MenB |
| PMB5371 | 0 | M | Blood | A166 | ST-269 complex | MenB |
| PMB5372 | 46 | M | CSF | B233 | ST-269 complex | MenB |
| PMB5373 | 58 | F | CSF | A22 | ST-41/44 complex/Lineage 3 | MenB |
| PMB5374 | 36 | F | Blood | A165 | ST-213 complex | MenB |
| PMB5375 | 0 | M | Blood | B24 | ST-32 complex/ET-5 complex | MenB |
| PMB5376 | 46 | F | Blood | A05 | ST-213 complex | MenB |
| PMB5377 | 0 | F | Blood | A22 | ST-41/44 complex/Lineage 3 | MenB |
| PMB5378 | 28 | M | CSF | A22 | ST-103 complex | MenC |
| PMB5379 | 67 | M | Blood | A19 | ST-5436 | MenY |
| PMB5380 | 14 | M | Blood | B09 | ST-1157 complex | MenB |
| PMB5381 | 1 | F | Blood | B133 | ST-32 complex/ET-5 complex | MenB |
| PMB5382 | 52 | F | Blood | A19 | ST-5436 | MenY |
| PMB5383 | 4 | F | CSF | A06 | ST-461 complex | MenB |
| PMB5384 | 20 | M | CSF | A154 | ST-1575 | MenB |
| PMB5385 | 0 | F | Joint | A15 | ST-23 complex/Cluster A3 | MenY |
| PMB5386 | 46 | F | CSF | B03 | ST-41/44 complex/Lineage 3 | MenB |
| PMB5387 | 16 | F | Blood | B24 | ST-32 complex/ET-5 complex | MenB |
| PMB5388 | 0 | M | CSF | B24 | ST-32 complex/ET-5 complex | MenB |
| PMB5389 | 0 | M | CSF | A22 | ST-41/44 complex/Lineage 3 | MenB |
| PMB5390 | 0 | M | CSF | B133 | ST-32 complex/ET-5 complex | MenB |
| PMB5391 | 13 | M | Blood | B209 | ST-167 complex | MenY |
| PMB5392 | 69 | F | Blood | A22 | ST-269 complex | MenB |
| PMB5393 | 54 | M | Joint | A22 | ST-41/44 complex/Lineage 3 | MenB |
| PMB5394 | 1 | F | Blood | B09 | ST-1157 complex | MenB |
| PMB5395 | 16 | F | Blood | A10 | ST-11 complex/ET-37 complex | MenW |
| PMB5396 | 25 | F | Blood | B16 | ST-41/44 complex/Lineage 3 | MenB |
| PMB5397 | 1 | F | Blood | B24 | ST-32 complex/ET-5 complex | MenB |
| PMB5398 | 86 | M | Blood | A12 | ST-167 complex | MenY |
| PMB5399 | 0 | F | CSF | B03 | ST-41/44 complex/Lineage 3 | MenB |
| PMB5400 | 3 | F | CSF | B03 | ST-41/44 complex/Lineage 3 | MenB |
| PMB5401 | 22 | F | CSF | B44 | ST-269 complex | MenB |
| PMB5402 | 65 | M | Blood | A15 | ST-23 complex/Cluster A3 | MenY |
| PMB5403 | 86 | F | Blood | A07 | ST-92 complex | MenY |
| PMB5404 | 69 | M | Blood | A15 | ST-23 complex/Cluster A3 | MenY |
| PMB5405 | 10 | M | Blood | A05 | ST-213 complex | MenB |
| PMB5406 | 28 | F | Blood | A95 | ST-13632 | MenB |
| PMB5407 | 17 | M | CSF | A22 | ST-41/44 complex/Lineage 3 | MenB |
| PMB5408 | 54 | F | CSF | A22 | ST-269 complex | MenB |
| PMB5409 | 58 | M | Blood | B03 | ST-41/44 complex/Lineage 3 | MenB |
| PMB5410 | 22 | M | CSF | B228 | ST-41/44 complex/Lineage 3 | MenB |
| PMB5411 | 5 | M | CSF | B229 | ST-32 complex/ET-5 complex | MenB |
| PMB5412 | 39 | F | Blood | B24 | ST-32 complex/ET-5 complex | MenB |
| PMB5413 | 5 | F | Blood | B24 | ST-32 complex/ET-5 complex | MenB |
| PMB5414 | 0 | M | CSF | B24 | ST-32 complex/ET-5 complex | MenB |
| PMB5415 | 79 | F | Blood | A19 | ST-5436 | MenY |
| PMB5416 | 65 | F | CSF | B24 | ST-32 complex/ET-5 complex | MenB |
| PMB5417 | 10 | F | Blood | B24 | ST-32 complex/ET-5 complex | MenB |
| PMB5418 | 18 | F | Blood | B24 | ST-32 complex/ET-5 complex | MenB |
| PMB5419 | 78 | M | Blood | B03 | ST-41/44 complex/Lineage 3 | MenB |
| PMB5420 | 74 | M | Blood | A19 | ST-22 complex | MenW |
| PMB5421 | 90 | F | Blood | B03 | ST-41/44 complex/Lineage 3 | MenB |
| PMB5422 | 21 | F | Blood | B09 | ST-461 complex | MenB |
| PMB5423 | 2 | M | Blood | A167 | ST-41/44 complex/Lineage 3 | MenB |
| PMB5424 | 46 | M | Blood | B209 | ST-167 complex | MenY |
| PMB5425 | 90 | F | Blood | A158 | ST-2267 | MenC |
| PMB5426 | 18 | F | Blood | A91 | ST-41/44 complex/Lineage 3 | MenB |
| PMB5427 | 2 | M | Blood | A05 | ST-213 complex | MenB |
| PMB5428 | 0 | F | Blood | B47 | ST-213 complex | MenB |
| PMB5429 | 0 | F | CSF | B23 | ST-41/44 complex/Lineage 3 | MenB |
| PMB5430 | 9 | M | CSF | A07 | ST-162 complex | MenB |
| PMB5431 | 69 | F | Blood | A19 | ST-22 complex | MenW |
| PMB5432 | 58 | M | Blood | B03 | ST-41/44 complex/Lineage 3 | MenB |
| PMB5433 | 58 | F | Blood | B133 | ST-32 complex/ET-5 complex | MenB |
| PMB5434 | 58 | F | CSF | B44 | ST-269 complex | MenB |
| PMB5435 | 0 | M | CSF | A22 | ST-41/44 complex/Lineage 3 | MenB |
| PMB5436 | 71 | F | Blood | A19 | ST-5436 | MenY |
| PMB5437 | 61 | F | Blood | B03 | ST-41/44 complex/Lineage 3 | MenB |
| PMB5438 | 51 | M | Blood | B03 | ST-41/44 complex/Lineage 3 | MenB |
| PMB5439 | 93 | M | Blood | A22 | ST-41/44 complex/Lineage 3 | MenB |
| PMB5440 | 17 | M | Blood | B09 | ST-60 complex | MenE |
| PMB5441 | 2 | M | Blood | A22 | ST-3934 | MenB |
| PMB5442 | 6 | M | Skin | B03 | ST-41/44 complex/Lineage 3 | MenB |
| PMB5443 | 17 | M | CSF | B03 | ST-41/44 complex/Lineage 3 | MenB |
| PMB5444 | 77 | F | Blood | A10 | ST-11 complex/ET-37 complex | MenC |
| PMB5445 | 1 | M | CSF | A22 | ST-269 complex | MenB |
| PMB5446 | 1 | F | CSF | B24 | ST-32 complex/ET-5 complex | MenB |
| PMB5447 | 0 | F | Blood | B03 | ST-41/44 complex/Lineage 3 | MenB |
| PMB5448 | 3 | F | Blood | B03 | ST-41/44 complex/Lineage 3 | MenB |
| PMB5449 | 5 | M | Blood | A22 | ST-41/44 complex/Lineage 3 | MenB |
| PMB5450 | 0 | M | CSF | B133 | ST-32 complex/ET-5 complex | MenB |
| PMB5451 | 17 | M | CSF | B133 | ST-32 complex/ET-5 complex | MenB |
| PMB5452 | 4 | M | CSF | B133 | ST-32 complex/ET-5 complex | MenB |
| PMB5453 | 2 | F | Blood | A22 | ST-41/44 complex/Lineage 3 | MenB |
| PMB5454 | 7 | M | Blood | B03 | ST-41/44 complex/Lineage 3 | MenB |
| PMB5455 | 71 | M | Blood | A19 | ST-5436 | MenY |
| PMB5456 | 74 | F | Blood | A22 | ST-269 complex | MenB |
| PMB5457 | UNK | M | Blood | B24 | ST-32 complex/ET-5 complex | MenB |
| PMB5458 | 15 | M | Blood | B09 | ST-60 complex | MenE |
| PMB5459 | 0 | M | Blood | B231 | ST-162 complex | MenB |
| PMB5460 | 66 | F | Blood | B03 | ST-41/44 complex/Lineage 3 | MenB |
| PMB5461 | 17 | F | Blood | B24 | ST-32 complex/ET-5 complex | MenB |
| PMB5462 | 18 | M | CSF | B133 | ST-32 complex/ET-5 complex | MenB |
| PMB5463 | 54 | F | CSF | B03 | ST-41/44 complex/Lineage 3 | MenB |
| PMB5464 | 5 | M | CSF | B03 | ST-41/44 complex/Lineage 3 | MenB |
| PMB5465 | 1 | M | CSF | A05 | ST-213 complex | MenB |
| PMB5466 | 0 | M | CSF | B03 | ST-41/44 complex/Lineage 3 | MenB |
| PMB5467 | 60 | M | Blood | B09 | ST-1157 complex | MenX |
| PMB5468 | 16 | M | CSF | B73 | ST-269 complex | MenB |
| PMB5469 | 7 | M | Blood | B03 | ST-41/44 complex/Lineage 3 | MenB |
| PMB5470 | 54 | M | Blood | A07 | ST-162 complex | MenB |
| PMB5471 | 3 | F | CSF | A164 | ST-213 complex | MenB |
| PMB5472 | 0 | F | CSF | B03 | ST-41/44 complex/Lineage 3 | MenB |
| PMB5473 | 18 | F | Blood | B24 | ST-32 complex/ET-5 complex | MenB |
| PMB5474 | 18 | F | Blood | A15 | ST-23 complex/Cluster A3 | MenY |
| PMB5475 | 50 | F | Blood | B03 | ST-41/44 complex/Lineage 3 | MenC |
| PMB5476 | 25 | M | Blood | A07 | ST-174 complex | MenY |
| PMB5477 | 24 | F | Blood | B24 | ST-32 complex/ET-5 complex | MenB |
| PMB5478 | 0 | F | CSF | A47 | ST-213 complex | MenB |
| PMB5479 | 79 | F | Blood | A19 | ST-5436 | MenY |
| PMB5480 | 0 | M | CSF | A22 | ST-269 complex | MenB |
| PMB5481 | 84 | M | Blood | A26 | ST-167 complex | MenY |
| PMB5482 | 58 | M | Blood | B24 | ST-32 complex/ET-5 complex | MenB |
| PMB5483 | 1 | F | CSF | B133 | ST-32 complex/ET-5 complex | MenB |
| PMB5484 | 88 | F | Blood | A15 | ST-23 complex/Cluster A3 | MenY |
| PMB5485 | 7 | F | Blood | B44 | ST-269 complex | MenB |
| PMB5486 | 5 | M | Blood | B44 | ST-269 complex | MenB |
| PMB5487 | 2 | M | Blood | B24 | ST-32 complex/ET-5 complex | MenB |
| PMB5488 | 60 | M | CSF | B133 | ST-32 complex/ET-5 complex | MenB |
| PMB5489 | 78 | F | Blood | B03 | ST-41/44 complex/Lineage 3 | MenB |
| PMB5490 | 29 | F | CSF | A15 | ST-23 complex/Cluster A3 | MenY |
| PMB5491 | 3 | F | CSF | B03 | ST-41/44 complex/Lineage 3 | MenB |
| PMB5492 | 9 | M | Blood | A19 | ST-5436 | MenY |
| PMB5493 | 45 | M | Blood | B44 | ST-269 complex | MenB |
| PMB5494 | 0 | M | CSF | A10 | ST-11 complex/ET-37 complex | MenW |
| PMB5495 | 38 | F | Blood | B44 | ST-269 complex | MenB |
| PMB5496 | 18 | M | Blood | A91 | ST-41/44 complex/Lineage 3 | MenB |
| PMB5497 | 3 | M | Blood | B24 | ST-32 complex/ET-5 complex | MenB |
| PMB5498 | 70 | F | Blood | A15 | ST-23 complex/Cluster A3 | MenY |
| PMB5499 | 17 | F | CSF | A05 | ST-213 complex | MenB |
| PMB5500 | 9 | F | CSF | B24 | ST-32 complex/ET-5 complex | MenB |
| PMB5501 | 24 | M | Blood | A62 | ST-5932 | MenC |
| PMB5502 | 55 | F | Blood | A19 | ST-22 complex | MenW |
| PMB5503 | 1 | F | CSF | B24 | ST-32 complex/ET-5 complex | MenB |
| PMB5504 | 17 | M | CSF | B133 | ST-32 complex/ET-5 complex | MenB |
| PMB5505 | 0 | F | CSF | A171 | ST-213 complex | MenB |
| PMB5506 | 1 | F | Blood | B254 | ST-1575 | MenB |
| PMB5507 | 3 | F | CSF | B133 | ST-32 complex/ET-5 complex | MenB |
| PMB5508 | 2 | F | Blood | B230 | ST-1111 | MenB |
| PMB5509 | 28 | F | Blood | B03 | ST-41/44 complex/Lineage 3 | MenB |
| PMB5510 | 52 | F | CSF | B09 | ST-1157 complex | MenX |
| PMB5511 | 37 | M | CSF | A166 | ST-2003 | MenB |
| PMB5512 | 70 | F | CSF | A10 | ST-11 complex/ET-37 complex | MenC |
| PMB5513 | 19 | M | CSF | A15 | ST-23 complex/Cluster A3 | MenY |
| PMB5514 | 8 | F | Blood | B03 | ST-41/44 complex/Lineage 3 | MenB |
| PMB5515 | 61 | F | Blood | A19 | ST-7952 | MenY |
| PMB5516 | 17 | M | CSF | A168 | ST-213 complex | MenB |
| PMB5517 | 36 | M | Blood | B24 | ST-32 complex/ET-5 complex | MenB |
