# Supplementary figures and images for: Genotypic and phenotypic comparison of Neisseria meningitidis carriage and invasive disease isolates contemporaneously collected in the Netherlands
Source: FEMS Microbiol Lett. 2025 Dec 16;373:fnaf140. doi: 10.1093/femsle/fnaf140 (PMC12794619; doi:10.1093/femsle/fnaf140)

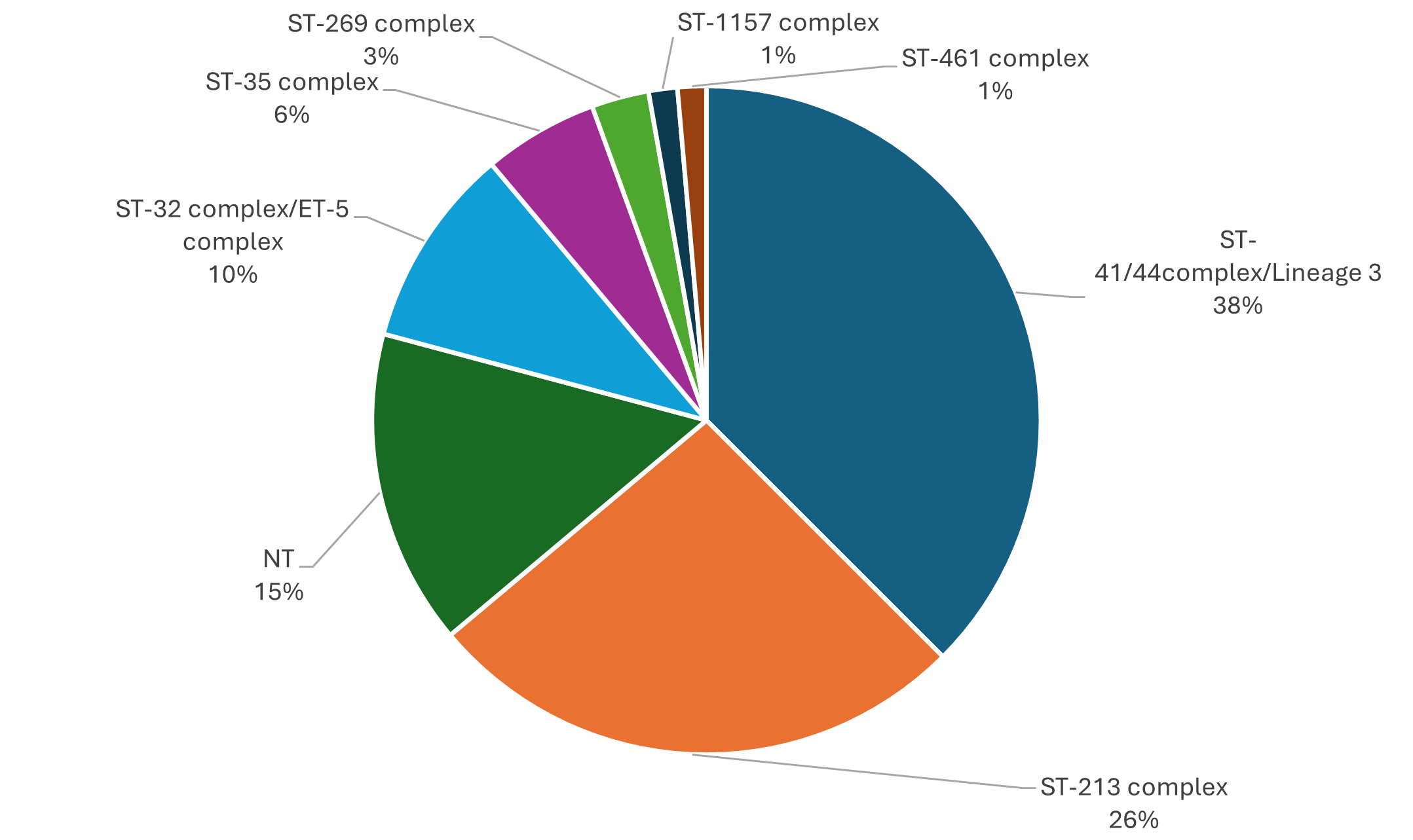

Supplement: fnaf140_Supplemental_Files [file fnaf140_supplemental_files.zip › Supplementary Figure 1A.tif]

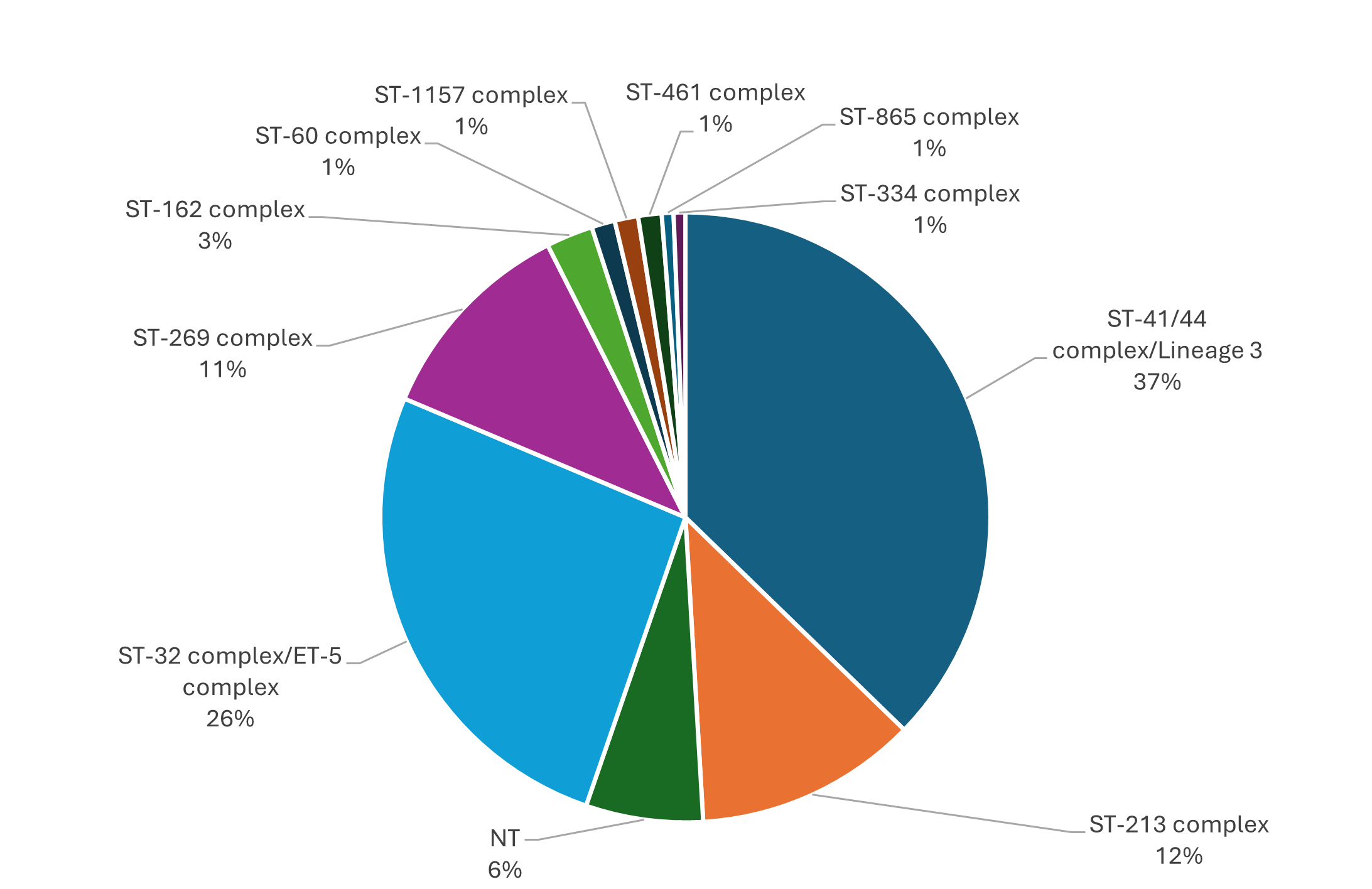

Supplement: fnaf140_Supplemental_Files [file fnaf140_supplemental_files.zip › Supplementary Figure 1B.tif]

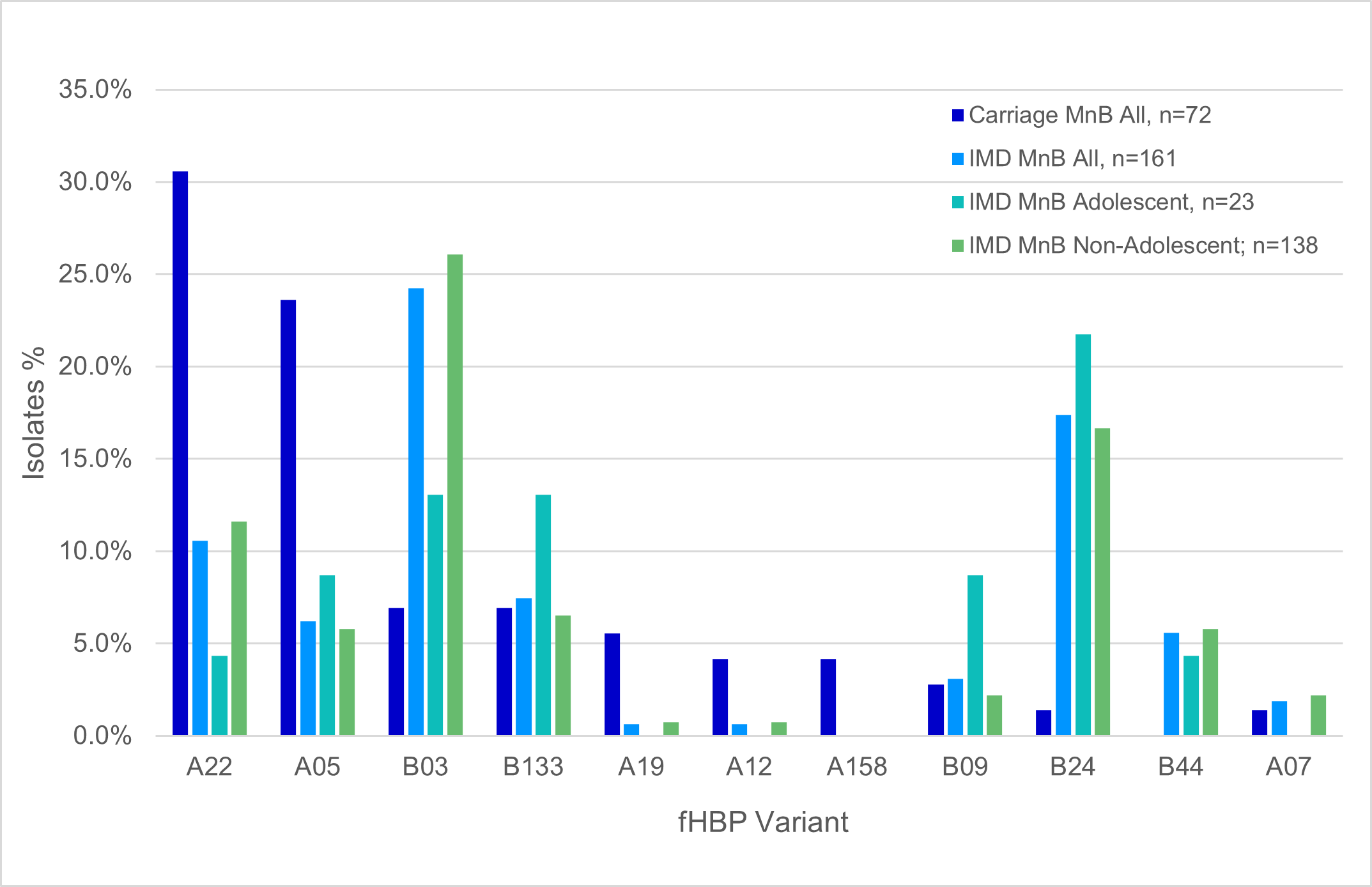

Supplement: fnaf140_Supplemental_Files [file fnaf140_supplemental_files.zip › Supplementary Figure 2.tif]

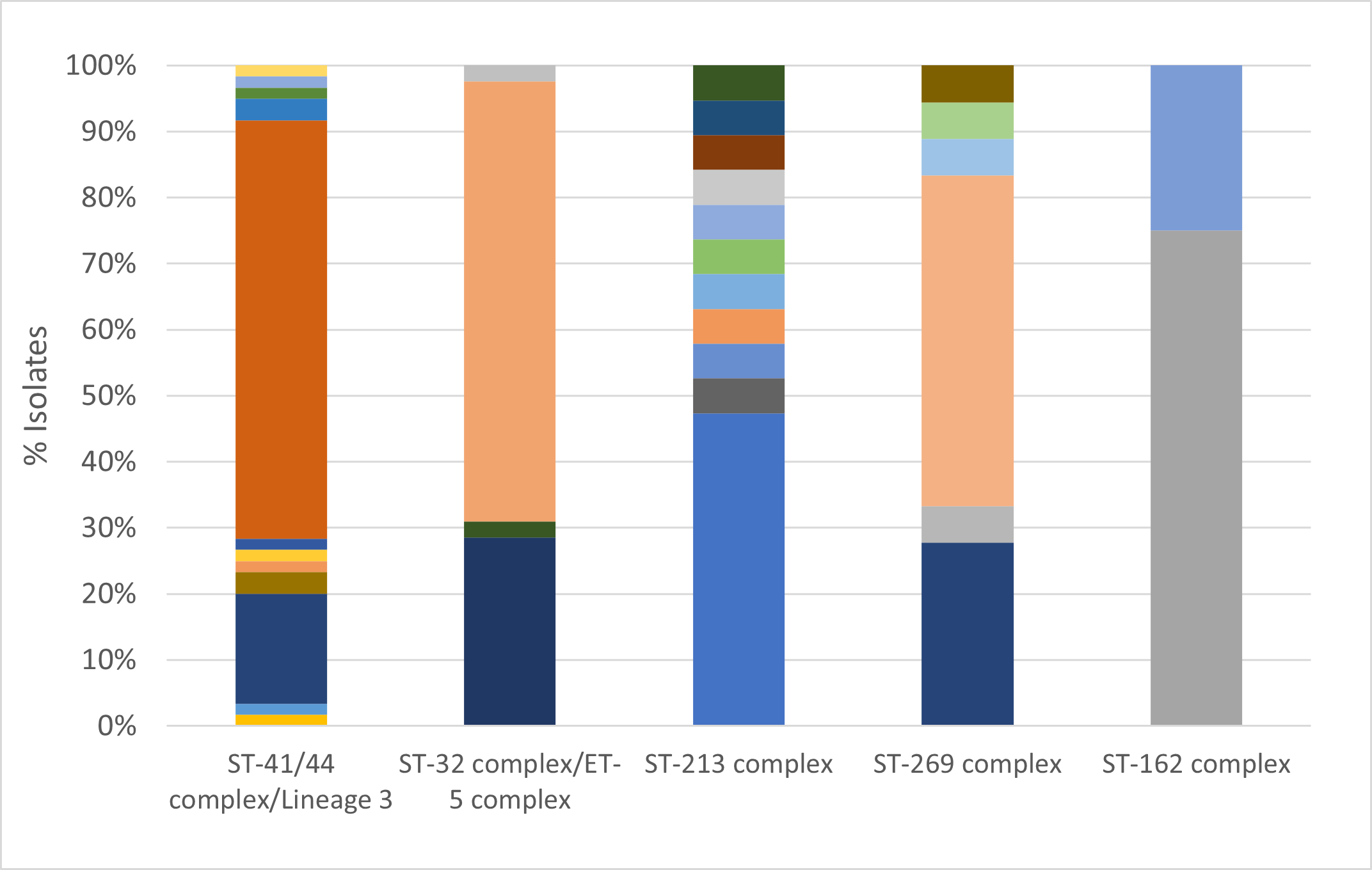

Supplement: fnaf140_Supplemental_Files [file fnaf140_supplemental_files.zip › Supplementary Figure 3A.tif]

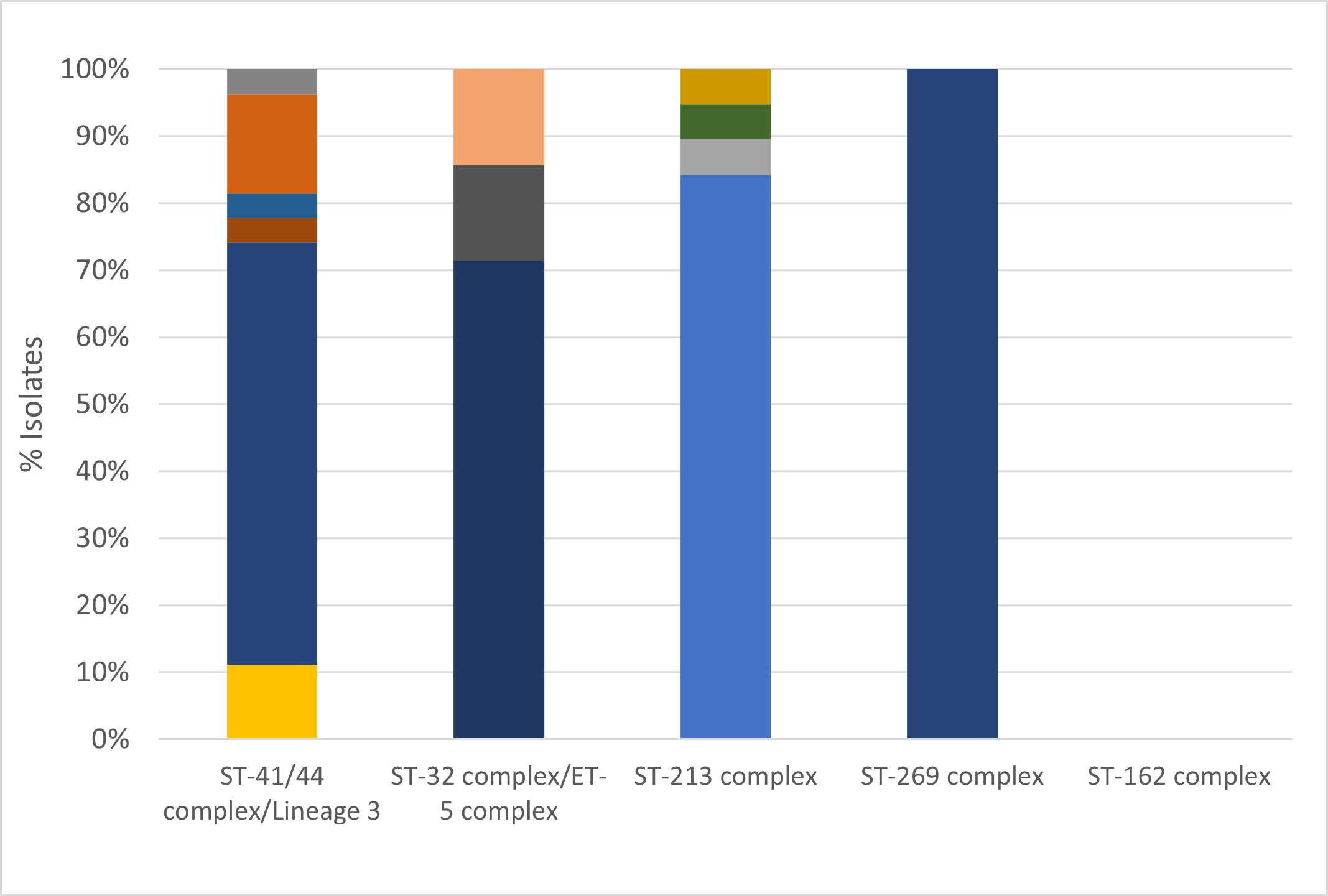

Supplement: fnaf140_Supplemental_Files [file fnaf140_supplemental_files.zip › Supplementary Figure 3B.tif]

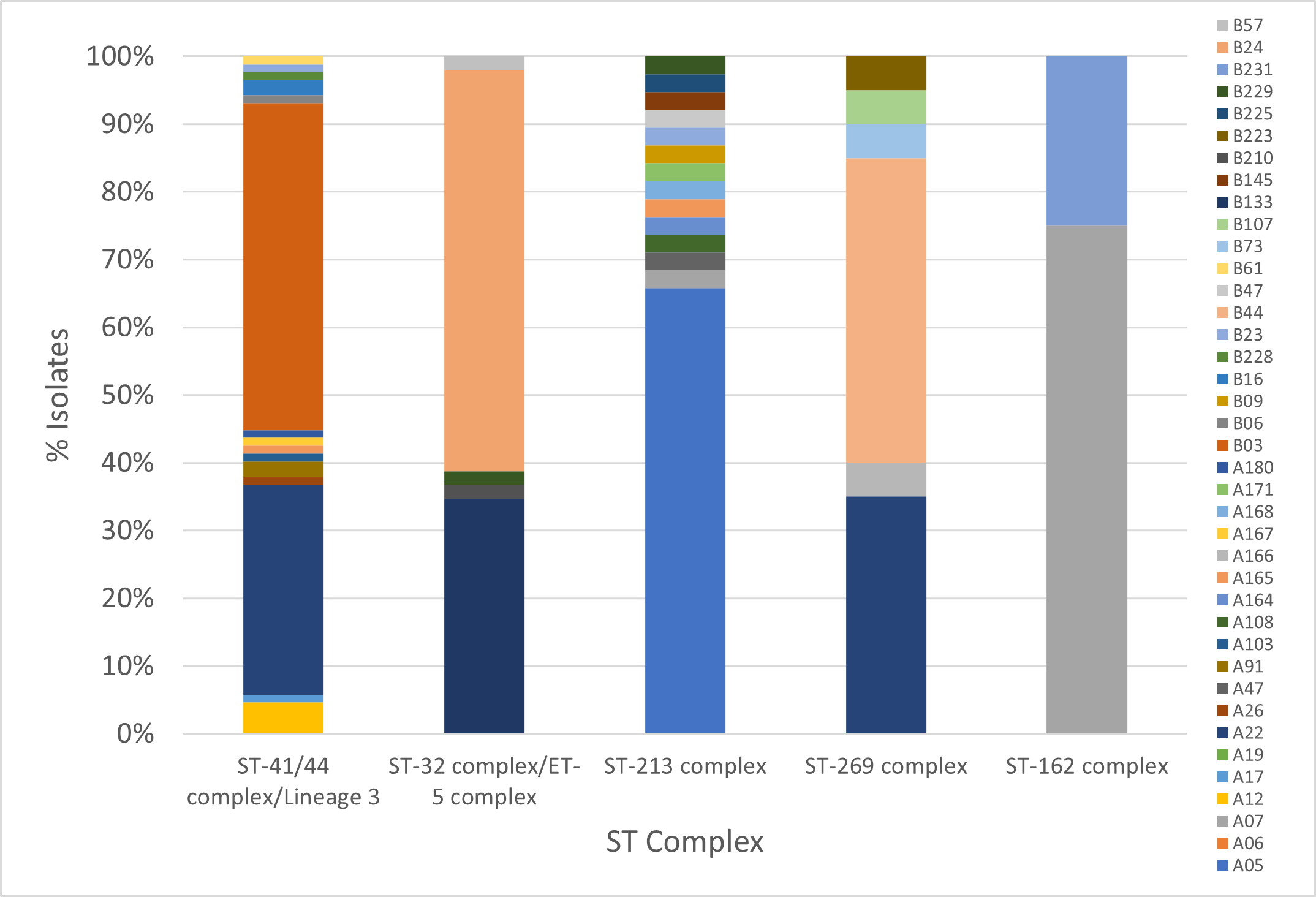

Supplement: fnaf140_Supplemental_Files [file fnaf140_supplemental_files.zip › Supplementary Figure 3C.tif]

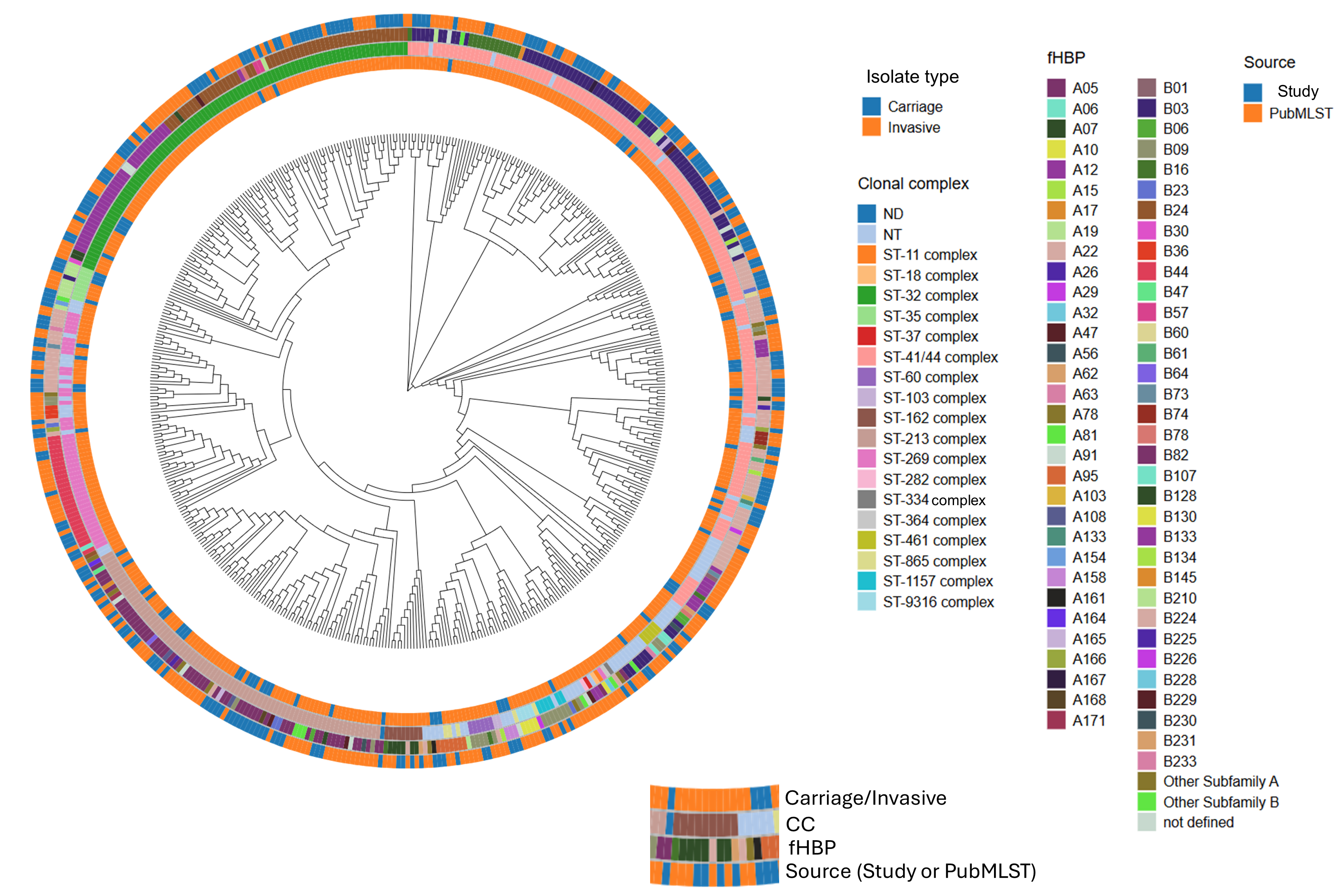

Supplement: fnaf140_Supplemental_Files [file fnaf140_supplemental_files.zip › Supplementary Figure 4.tif]

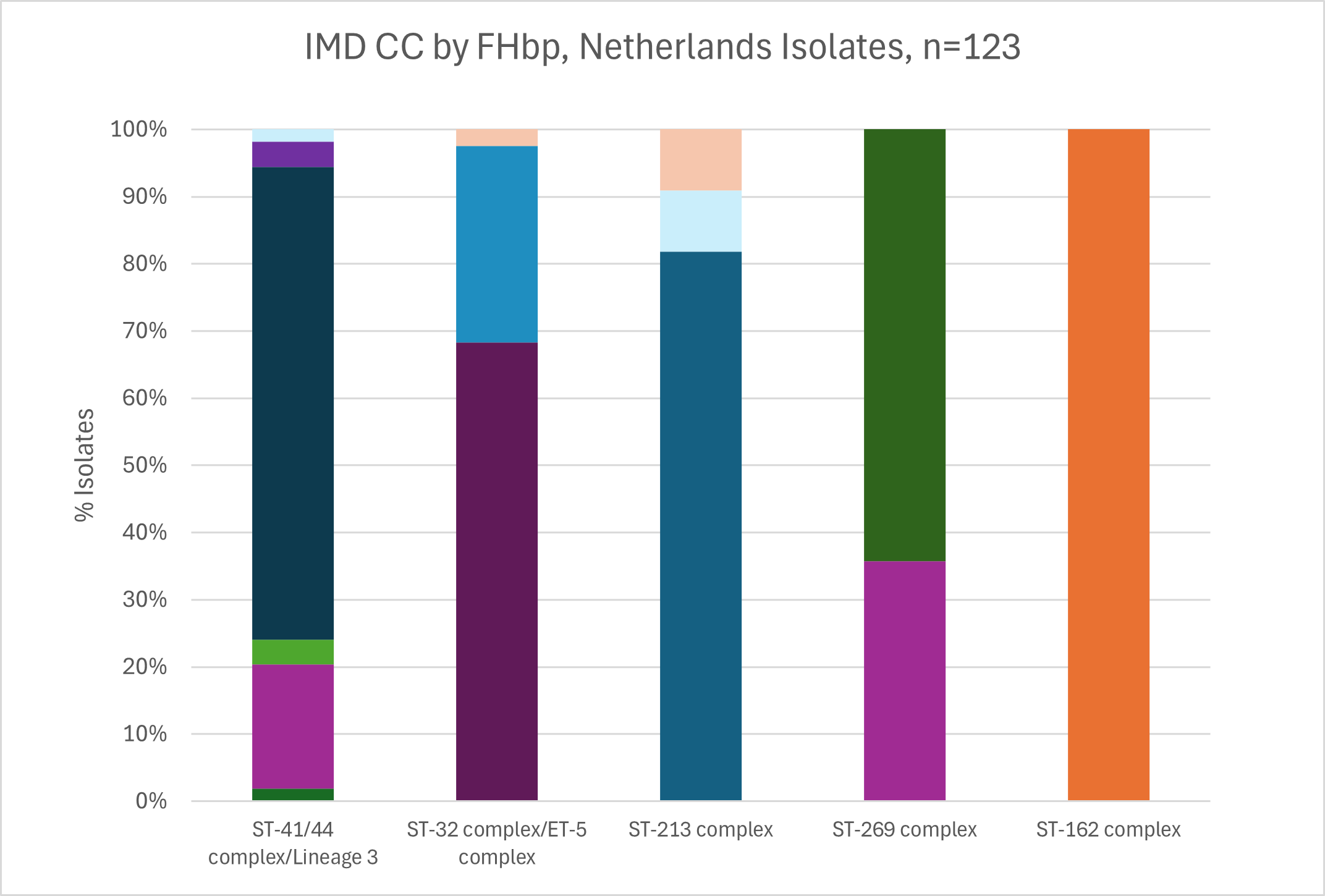

Supplement: fnaf140_Supplemental_Files [file fnaf140_supplemental_files.zip › Supplementary Figure 5A.tif]

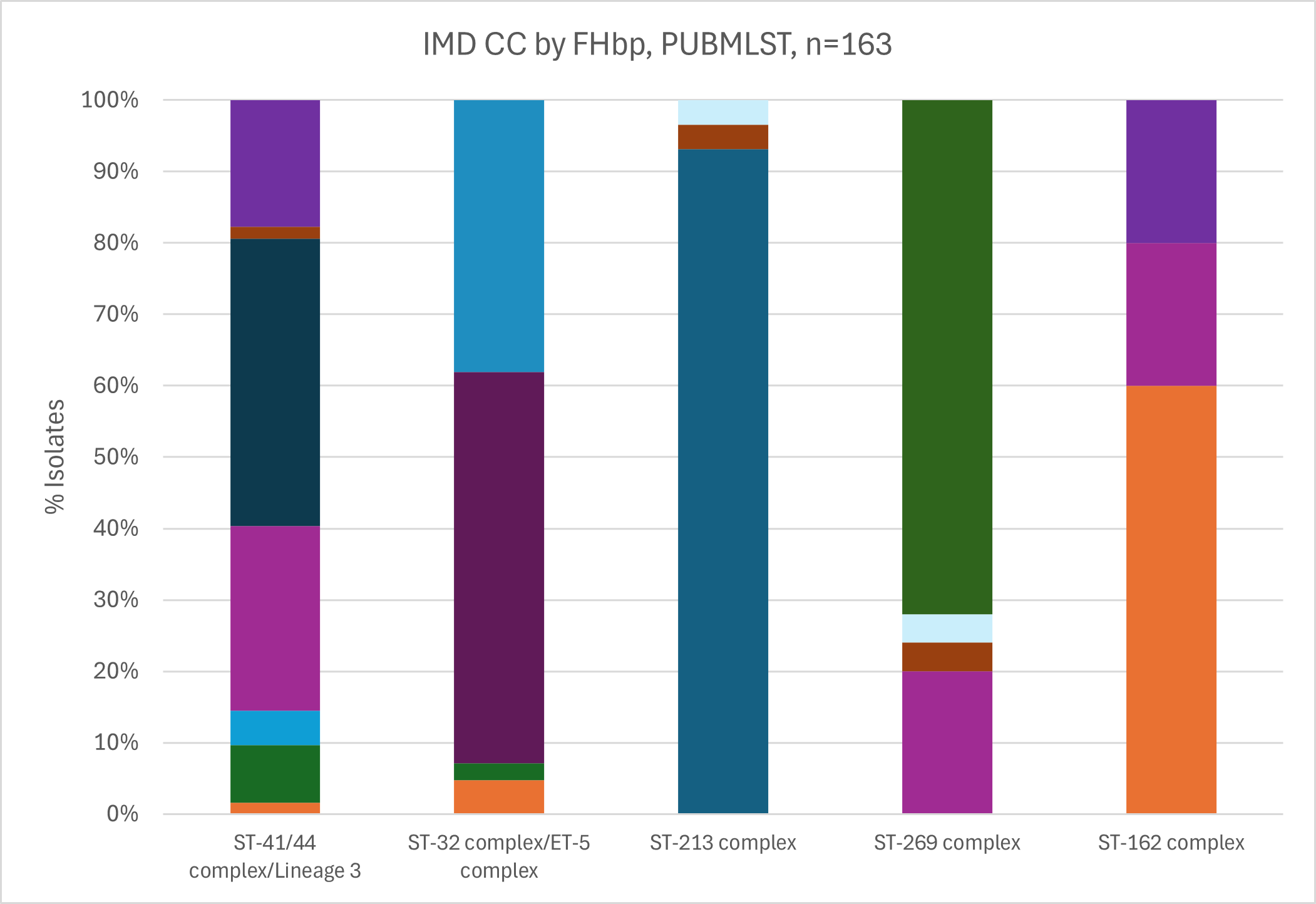

Supplement: fnaf140_Supplemental_Files [file fnaf140_supplemental_files.zip › Supplementary Figure 5B.tif]

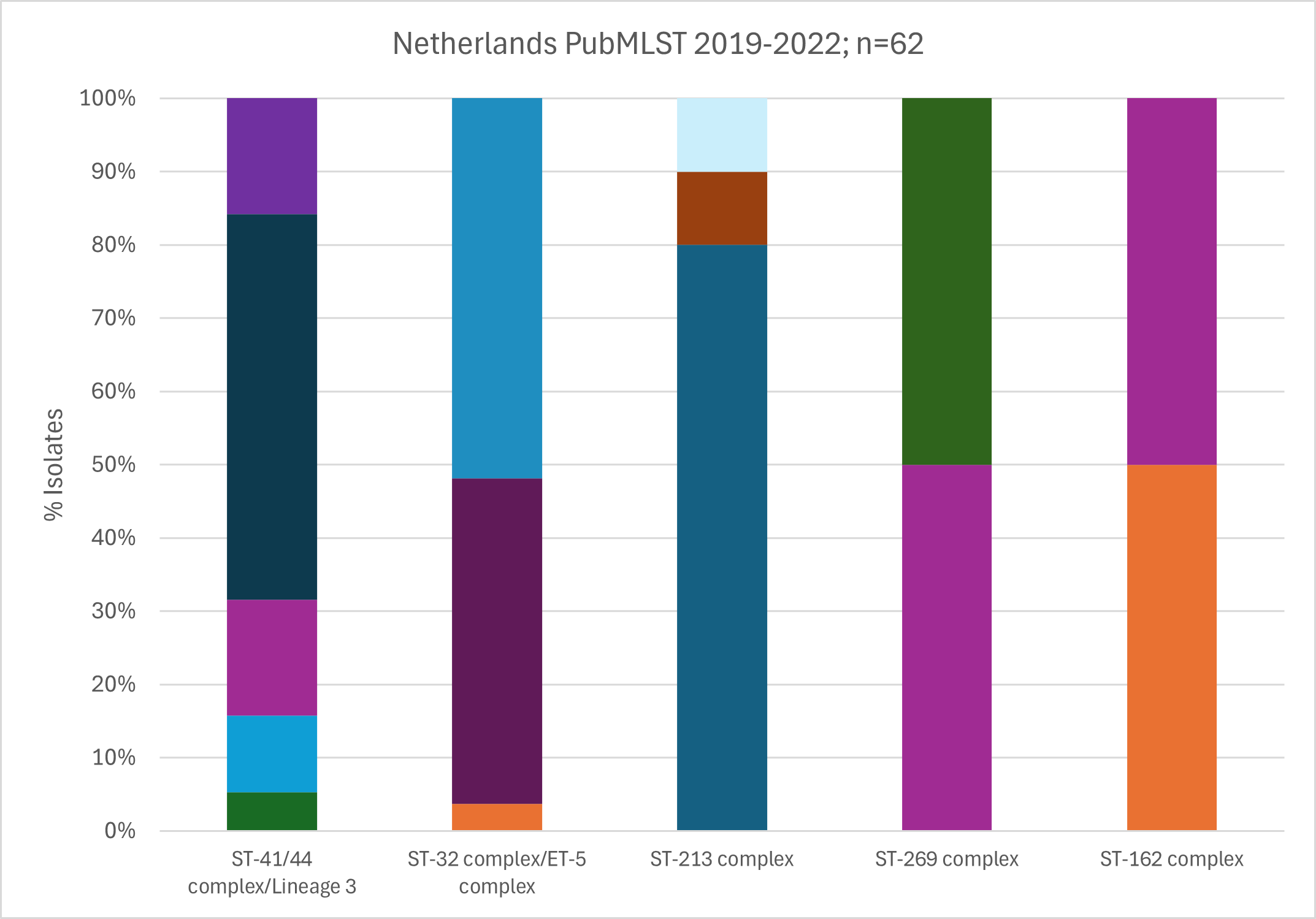

Supplement: fnaf140_Supplemental_Files [file fnaf140_supplemental_files.zip › Supplementary Figure 5C.tif]

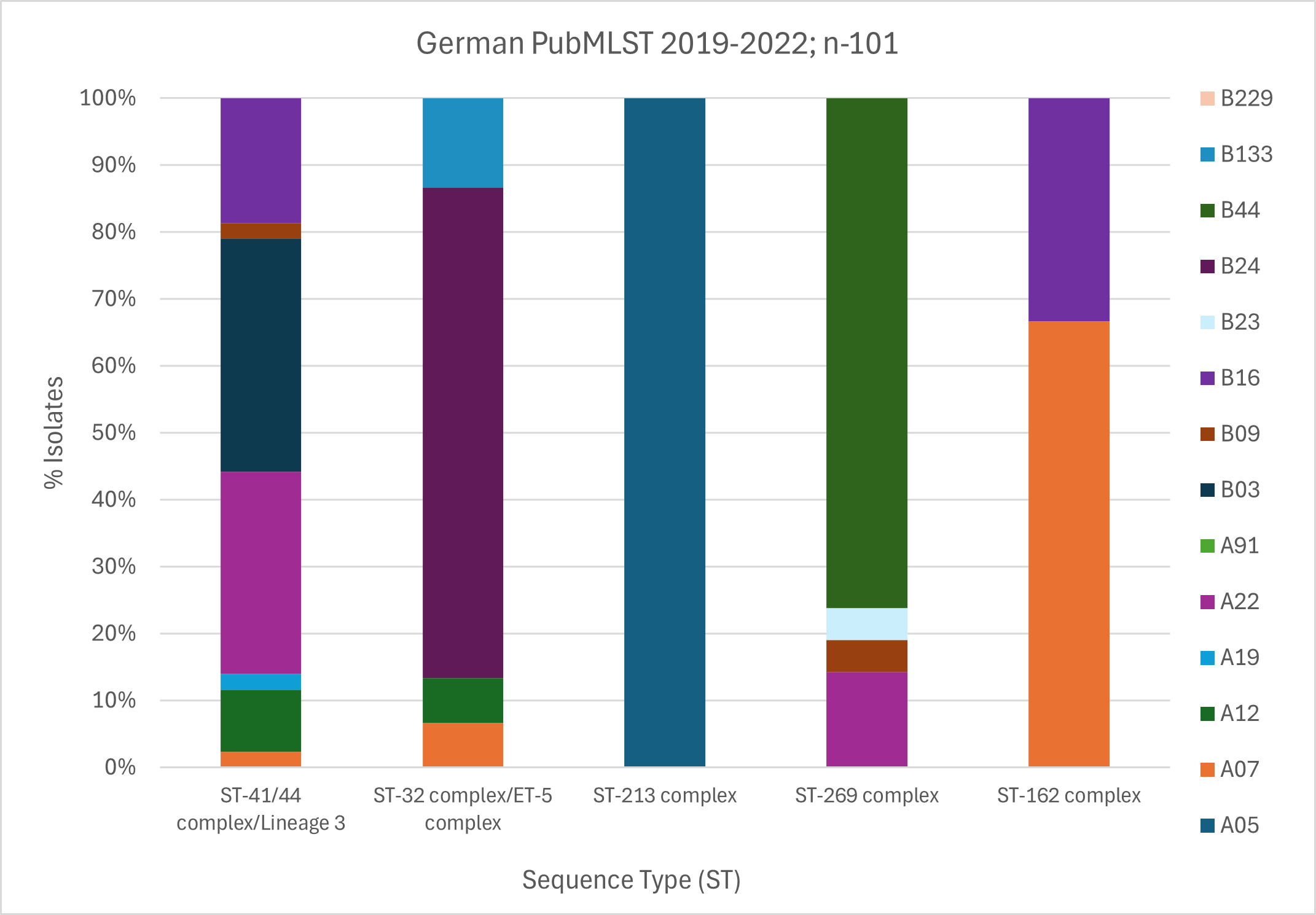

Supplement: fnaf140_Supplemental_Files [file fnaf140_supplemental_files.zip › Supplementary Figure 5D.tif]
